# Alkaline Leaching: A Facile Surface Activation Strategy to Improve the Reactivity of Air Electrodes for Solid Oxide Fuel Cells

**DOI:** 10.1002/adma.202511053

**Published:** 2026-01-30

**Authors:** Yeongtaek Hong, Hyunseung Kim, Sang Won Lee, Yong Beom Kim, SungHyun Jeon, Sangwoo Kim, Hainan Sun, Jeongah Lee, Seongwoo Nam, Seungwoo Roh, Tae Ho Shin, WooChul Jung

**Affiliations:** ^1^ Department of Materials Science and Engineering Seoul National University (SNU) Seoul Republic of Korea; ^2^ Research Institute of Advanced Materials (RIAM) Seoul National University (SNU) Seoul Republic of Korea; ^3^ Energy Research Institute @ NTU (ERI@N) Interdisciplinary Graduate School Nanyang Technological University Singapore; ^4^ Korea Institute of Ceramic Engineering and Technology (KICET) Jinju Republic of Korea; ^5^ Department of Materials Science and Engineering Northwestern University Evanston Illinois USA; ^6^ Department of Materials Science and Engineering Korea Advanced Institute of Science and Technology (KAIST) Daejeon Republic of Korea; ^7^ Platform Technology Research Center LG Chem Republic of Korea; ^8^ School of Chemistry and Chemical Engineering Nantong University Jiangsu P. R. China

**Keywords:** air electrodes, alkaline leaching, perovskite oxides, solid oxide fuel cells, surface modification

## Abstract

The energy conversion efficiency of solid oxide fuel cells is primarily governed by the performance of their air electrodes. Several surface modification techniques, including nanocatalyst decoration, surface coating, and acid etching, have been reported to enhance the performance of air electrodes. However, these approaches often face limitations in cost and time efficiency. In this study, we propose alkaline leaching as a straightforward and innovative strategy to activate the surface of mixed‐conducting oxides by selectively dissolving the A‐site cation during bias application in an alkaline solution. After 10 min of alkaline leaching, the surface of the PrBa_0.8_Ca_0.2_Co_2_O_5+δ_ electrode becomes cobalt‐rich and amorphous, recognized for its favorable impact on reactivity. As a result, when the surface‐modified electrode is used as the air electrode in a solid oxide fuel cell, it exhibits a 5.6 fold enhancement in catalytic activity, achieving an area‐specific resistance of 0.019 Ω cm^2^. Single cell measurements further demonstrate a 33 % increase in maximum power density, reaching 2.10 W cm^−2^ at 650°C. This work provides a strategic approach for engineering highly active oxide surfaces, leveraging a straightforward system operable at ambient pressure and room temperature, with broad applicability across diverse devices.

## Graphical abstract

This study shows that alkaline leaching of PrBa_0.8_Ca_0.2_Co_2_O_5+δ_ under anodic bias selectively removes Ba and Ca, forming a cobalt‐rich and amorphous layer. The modified surface significantly boosts ORR activity, offering a simple and effective strategy to improve electrochemical device performance.

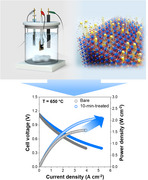

## 1 Introduction

Solid oxide fuel cells (SOFCs) hold great promise as next‐generation energy conversion devices, owing to their exceptional conversion efficiency [1, 2, 3, 4, 5]. Nonetheless, given their high operating temperatures (>750°C), they pose certain challenges, including limited material selection, elevated system cost, and thermal degradation. Consequently, significant efforts have been directed toward reducing the operating temperature of SOFCs [6, 7, 8, 9]. However, at lower temperatures (<650°C), the oxygen reduction reaction (ORR) notably slows down due to the high activation energy associated with the commonly used air electrode materials [10, 11, 12]. Previous studies have primarily focused on the materials for air electrodes to enhance performance at low temperatures. However, there remains a demand for innovative strategies that can improve performance and be directly applied to commercialized systems.

In this context, researchers have explored various modification techniques for electrode surfaces. These methods aim to create alternative reaction pathways with reduced activation barriers for ORR or to eliminate deteriorated or passivated surface components. Examples include decorating with nanocatalysts through infiltration [13, 14, 15], coating metal or metal oxides on the surface using atomic layer deposition [16, 17, 18] or sputtering [19], and employing acid etching [20, 21]. For example, Namgung et al. achieved 2.7 fold enhanced electrode activity by infiltration of Sm_0.5_Sr_0.5_CoO_3‐δ_ nanoparticles onto the La_0.6_Sr_0.4_Co_0.2_Fe_0.8_O_3‐δ_ electrode [15], while Choi et al. coated La_1‐x_Sr_x_CoO_3‐δ_ on the surface of the La_0.6_Sr_0.4_Co_0.2_Fe_0.8_O_3‐δ_ air electrode of SOFCs using atomic layer deposition, resulting in a 1.8 fold increase in power density [17]. Additionally, Cai et al. obtained 4 fold enhanced activity by removing the inactive Sr‐segregated surface of the La_0.6_Sr_0.4_CoO_3‐δ_ air electrode via HCl etching [21]. However, the nanocatalyst decoration methods require tedious heat treatment steps [22], and surface coating approaches necessitate complicated vacuum systems [10]. In the case of acid etching, there are limitations in controlling surface composition and the risk of acid‐induced corrosion in other compartments of SOFCs, including NiO [23] in the fuel electrode and the interconnect metals [24]. To effectively enhance the surface of air electrodes, it is crucial to develop a method that is not only cost‐ and time‐efficient but also offers precise control over the process.

**FIGURE 1:**
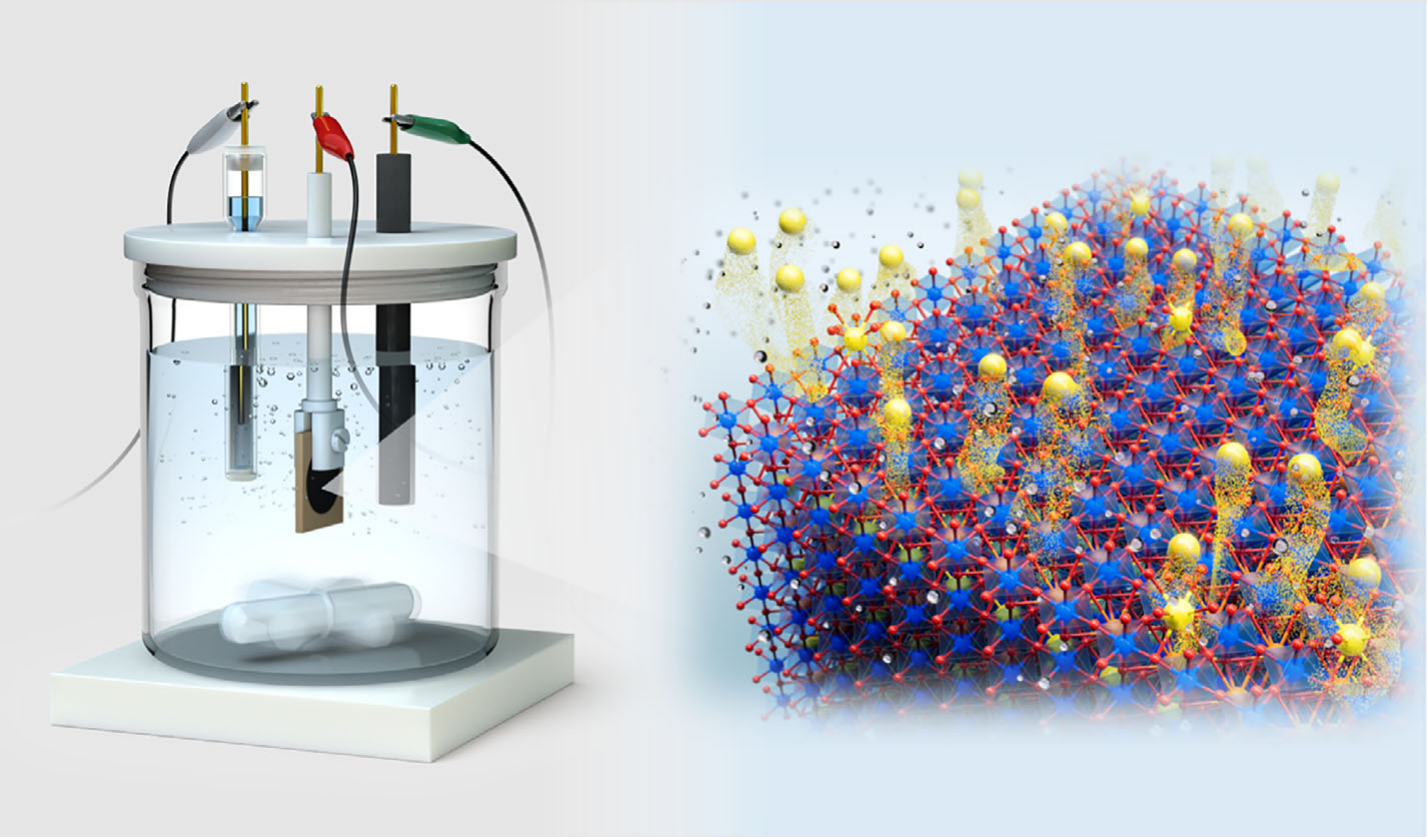
A schematic illustration of alkaline leaching as a surface modification strategy for PrBa_0.8_Ca_0.2_Co_2_O_5+δ_ (PBCC) electrodes. A conventional 3‐electrode system with the PBCC electrode as a working electrode and a graphite rod as a counter electrode is depicted on the left with 1 V (vs. Hg/HgO reference electrode) of constant anodic bias application at room temperature. During the treatment, alkaline earth metal cations, Ba^2+^ and Ca^2+^ in this case, are selectively dissolved in the 1 m KOH alkaline medium.

Here, we present a facile strategy to improve air electrode performance through surface modification utilizing alkaline leaching (Figure 1). Alkaline leaching refers to the process that occurs on the surface of oxides when exposed to an alkaline medium under anodic bias—a condition commonly encountered during the oxygen evolution reaction (OER) in alkaline water electrolysis [25, 26, 27, 28, 29, 30, 31, 32]. This process has been widely reported on conductive perovskite oxide‐based electrodes. As a result, the perovskite oxide surface undergoes reconstruction, characterized by the dissolution of A‐site alkaline earth metal cations, the exposure of B‐site transition metal (oxy)hydroxides, and the amorphization of the topmost surface [25, 33]. This leads to an amorphized surface enriched with B‐site transition metal species, which are known to be beneficial for the ORR [34, 35, 36, 37] under the operating conditions of SOFCs (>450°C). While alkaline leaching is expected to improve the performance of SOFC air electrodes, to the best of our knowledge, there have been no reports applying alkaline leaching to SOFC electrodes.

In this work, PrBa_0.8_Ca_0.2_Co_2_O_5+δ_ (PBCC) was selected as the air electrode material for the first case study to apply our strategy to SOFC electrode surfaces due to its superior ORR activity and long‐term stability [38, 39, 40, 41, 42, 43, 44, 45]. Alkaline leaching was applied directly to the symmetrical cell, which has the configuration of PBCC|Sm_0.2_Ce_0.8_O_2‐δ_ (SDC)|PBCC, under bias application in an alkaline solution at various times. Electrochemical impedance spectroscopy (EIS) was carried out to measure the ORR activity, which confirmed an area‐specific resistance of 0.019 Ω cm^2^ at 650°C, corresponding to a 5.6 fold increase in electrode activity only after 10 min of surface treatment compared to the pristine PBCC. The amorphous and cobalt‐rich electrode surface, known to be favorable for ORR reactivity [46, 47], was confirmed by high‐resolution transmission electron microscope (HRTEM) and energy‐dispersive X‐ray spectroscopy (EDS) analyses [50, 51]. Additionally, at the operating temperatures of SOFCs, the exposed cobalt‐rich layer spontaneously transformed into cobalt oxide particles [48, 49, 50, 51], which can act as additional catalytic sites for ORR [18, 52, 53, 54, 55]. X‐ray photoelectron spectroscopy (XPS) analysis revealed an increase in active oxygen species and a decrease in non‐lattice barium species, which have been identified as a cause of degraded activity [34]. To validate the practical applicability of our approach, alkaline leaching was directly applied to a single cell, resulting in a 2.10 W cm^−2^ of maximum power density at 650°C, corresponding to a 33 % improvement. Moreover, the treated cell maintained stable performance over 500 h of continuous operation. Our findings indicate that selective cation leaching, traditionally regarded as an unfavorable side effect of alkaline OER, can be leveraged as a straightforward technique to modulate surface chemistry and enhance the electrochemical reactivity of perovskite‐oxide‐based electrodes.

## 2 Results and Discussion

We validated the activity enhancement of the PBCC electrodes after the surface treatment by alkaline leaching. PBCC symmetrical cells with the configuration of PBCC|SDC|PBCC were treated in a 3‐electrode system, constructed with 1 m KOH solution (pH = 14) as an electrolyte and a graphite rod as the counter electrode. The PBCC electrode, serving as a working electrode, was contacted with a Pt clip. An anodic bias of 1 V between the reference and working electrodes (i.e., PBCC electrode) at room temperature (Figure 1).

### 2.1 Electrochemical Characterization

**FIGURE 2:**
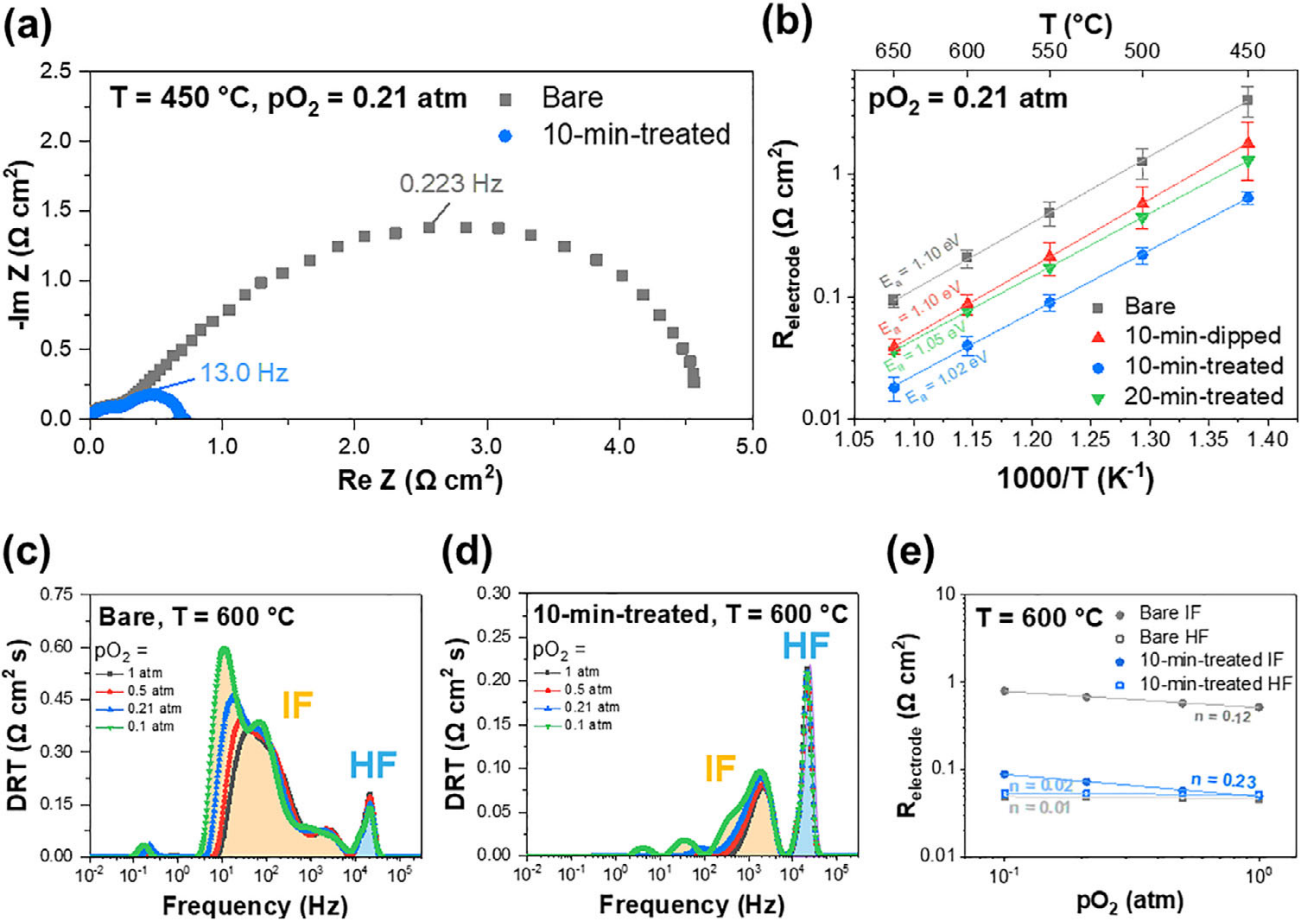
Enhanced electrochemical performance of PrBa_0.8_Ca_0.2_Co_2_O_5+δ_ (PBCC) electrodes via surface modification with alkaline leaching. (a) Impedance spectra of bare and 10 min‐treated PBCC symmetrical cells (PBCC|Sm_0.2_Ce_0.8_O_2‐δ_|PBCC) measured at *T* = 450°C. For ease of comparison, the offset resistance was omitted for each. (b) Arrhenius plots of electrode resistance (*R_electrode_*) fitted from the impedance spectra of bare, 10 min‐dipped, 10 min‐treated, and 20 min‐treated PBCC symmetrical cells. The graph was plotted using the average values obtained from three different cells, and the standard deviations are shown as error bars. Distribution of relaxation times (DRT) plots of (c) bare and (d) 10 min‐treated PBCC electrodes, deconvoluted to high‐frequency (HF) region and intermediate frequency (IF) region components. (e) *R_electrode_* calculated by integrating the area under DRT plots in each region, plotted as a function of *pO_2_*.

Alkaline leaching was performed for 10 and 20 min, and henceforth, each sample was denoted as ’10 min‐treated’ and ’20 min‐treated,’ respectively. Additionally, to observe the influence of bias application, a cell was dipped without bias in the same system for 10 min, denoted as ‘10 min‐dipped.’ Electrochemical impedance data from the EIS spectra were normalized by the area of the PBCC electrodes, and the Nyquist plots were obtained. In impedance spectra, the diameter of the semicircles indicates the electrode resistance; thus, a smaller size of the impedance spectra corresponds to a higher reactivity of electrodes. By comparing the EIS spectra of bare and 10 min‐treated PBCC symmetrical cells at 450°C (Figure 2a), a noticeable reduction in electrode resistance (4.80–0.64 Ω cm^2^) was observed after alkaline leaching, indicating a significant enhancement (7.5 fold) in reactivity.

Figure 2b shows the Arrhenius plot of electrode resistance, indicated as *R*_electrode_, as a function of the temperature. Each data point in the plot was obtained from the impedance spectra in the same manner as described in the previous paragraph. The most pronounced improvement in activity was observed for the PBCC electrode subjected to a 10 min alkaline leaching, resulting in a notable 10% reduction in the activation energy from 1.10 to 1.02 eV compared to the pristine PBCC electrode. The 10 min‐dipped PBCC electrode also exhibited a reduction of *R*_electrode_, which may be attributed to the removal of secondary barium‐rich phases on the surface of PBCC electrodes, which have an inert nature [34]. However, despite this activity improvement, the dipped electrode showed a larger *R*_electrode_ and a wider error range compared to the bias‐applied PBCC electrode. While the 20 min‐treated PBCC electrode also resulted in improved activity, the enhancement factor was less pronounced than that observed for the 10 min treatment.

Next, the electrode resistance before and after the surface treatment was quantitatively compared. Before the treatment, the electrode resistance of the bare PBCC electrode was measured to be 0.10, 0.24, 0.57, 1.5, and 4.9 Ω cm^2^ at 650°C, 600°C, 550°C, 500°C, and 450°C, respectively. After 10 min of alkaline leaching, the electrode resistance of the PBCC electrode was reduced to 0.019, 0.041, 0.094, 0.25, and 0.69 Ω cm^2^ at 650°C, 600°C, 550°C, 500°C, and 450°C, respectively, which were confirmed by the equivalent circuit fitting results as shown in Figure S1. The detailed fitting results are summarized in Table S1. The enhancement factor was calculated by dividing the electrode reactivity (reciprocal of the electrode resistance) of the leached sample by that of the bare sample (Figure S2). The enhancement of the ORR activity was 5.6 fold at 650°C, and notably 7.5 fold at 450°C, implying the advantage of our approach in terms of lowering operation temperatures of SOFCs. The enhancement factor by alkaline leaching is noteworthy compared to the previous surface modification techniques, as shown in Figure S3. The improved reactivity of the 10 min‐treated PBCC electrode remained at 550°C for 120 h without noticeable degradation (Figure S4).

These findings suggest that the application of an anodic bias during alkaline leaching appears to be more effective in enhancing electrochemical activity than dipping without bias. This may be due to the promotion of surface reconstruction under bias, leading to a more active surface for the ORR. The less pronounced enhancement observed in the 20 min‐treated PBCC electrode indicates that excessive cation dissolution may not further benefit the reactivity. This underscores the importance of optimizing the operation time of alkaline leaching to balance surface modification with structural integrity. The improvement of electrochemical activity is attributed to the enrichment of catalytically active transition metal cations at the electrode surface, which will be further discussed.

Distribution of relaxation time (DRT) analyses were conducted to further elucidate which steps in ORR were primarily affected by the surface treatment. Figure 2c,d demonstrates the DRT plots of bare and 10 min‐treated PBCC electrodes, respectively. The plots exhibit three distinctive regions–low frequency (LF), intermediate frequency (IF), and high frequency (HF)–with each region corresponding to the resistance of a specific electrochemical process. The LF peaks are less temperature dependent (Figure S5), suggesting that they may be associated with mass transfer processes such as gas diffusion [42, 56]. Since significant peaks arose in the IF and HF regions, while the LF component contribution to the total electrode resistance was negligible across all conditions, detailed analyses were focused on the IF and HF regions. The *pO_2_*‐dependencies of IF and HF resistance compartments were plotted in Figure 2e and were fitted with the following Equation 1 as in the previous study by Chen et al. [43]:

(1) $$R_{\mathrm{electrode}}\;=\;k{\left({\mathrm{pO}}_{2}\right)}^{-n}$$

The IF peaks, which are prominent components within *R*_electrode_, demonstrated a high *pO_2_*‐dependency (*n* = 0.12–0.23) and showed significant changes after alkaline leaching, in contrast to the HF peaks (*n* = 0.01–0.02). The *pO_2_*‐sensitive IF peaks notably decreased after the surface treatment, implying an improvement in the surface oxygen exchange reaction, for example, the dissociation and ionization process [43, 56].

A notable shift in the rate‐determining step of the surface oxygen exchange process was observed following the surface treatment, as evidenced by a change in the n‐value within the IF region from 0.12 to 0.23. Previous work by Rupp et al. has suggested that surface cobalt species provide the active site for the oxygen dissociation and ionization process [37]. The selective dissolution of alkaline earth metal cations during alkaline leaching resulted in increased exposure of these active cobalt sites compared to the pristine PBCC surface. Consequently, the increased *pO_2_*‐dependency of the electrode resistance can be attributed to a significantly accelerated rate of oxygen dissociation and ionization process.

### 2.2 Physicochemical Characterization

**FIGURE 3:**
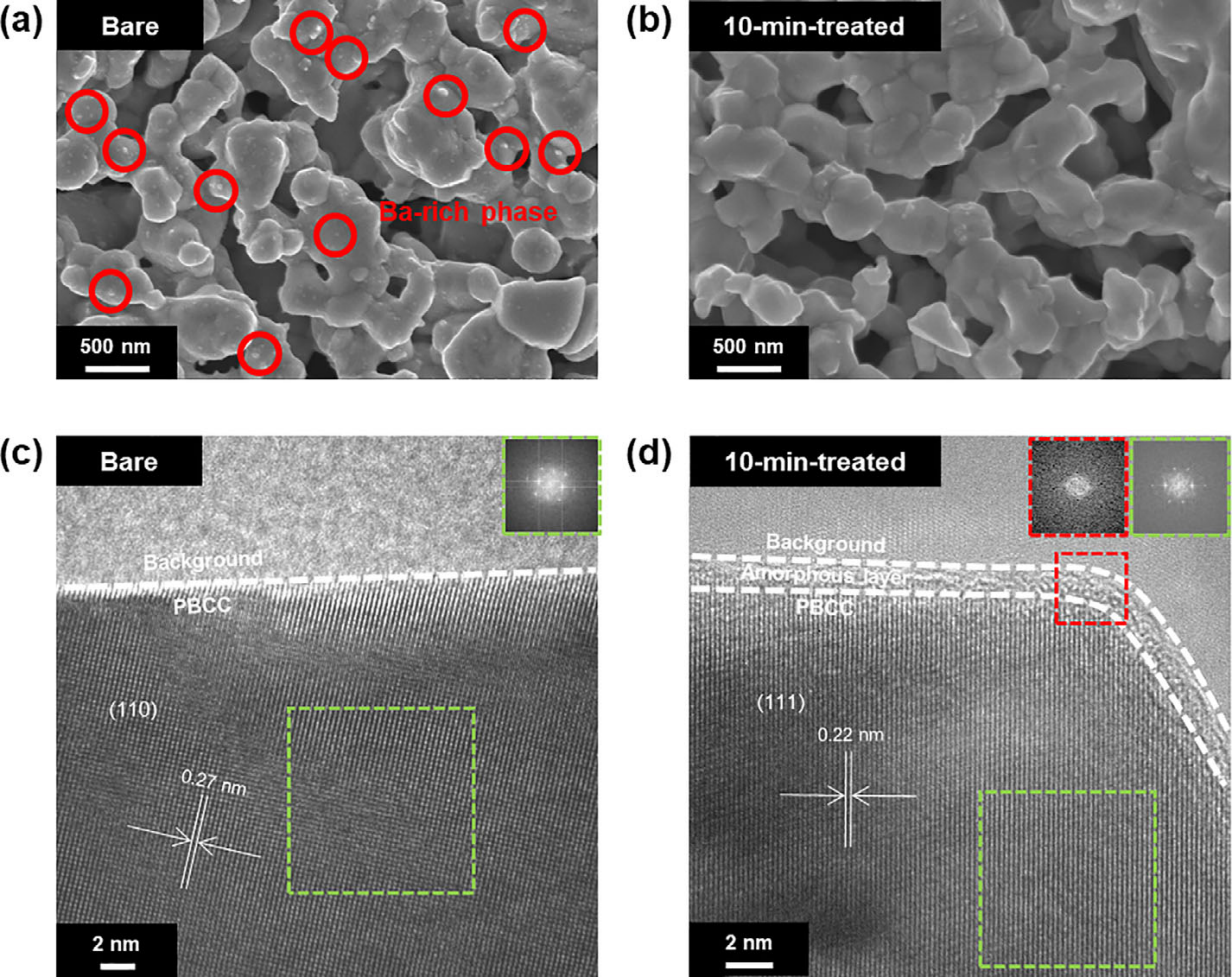
Scanning electron microscopy images of the (a) bare and (b) 10 min‐treated PrBa_0.8_Ca_0.2_Co_2_O_5+δ_ (PBCC) electrodes. Cross‐sectional high‐resolution transmission electron microscopy images of (c) bare and (d) 10 min‐treated PBCC electrodes. The corresponding fast Fourier transform images are shown as insets, color‐coded for consistency. The lattice spacing was calculated based on the indicated crystal planes of each PBCC electrode. An amorphous topmost surface layer was observed in the alkaline‐leaching‐treated PBCC electrode.

Scanning electron microscopy (SEM) images illustrate the microstructures of bare and 10 min‐treated PBCC electrodes, as illustrated in Figure 3a,b, respectively. The porous microstructure, consisting of continuously connected PBCC particles ranging from 500 nm to 1 µm, was clearly observed. It was found that the porous microstructures of PBCC electrodes remained unchanged after the alkaline leaching treatment. Before the surface treatment, distinctive surface particles, indicated as red circles in Figure 3a, were observed on the bare PBCC electrode. These particles were identified as barium‐rich secondary phases through TEM‐EDS analysis (Figure S6), which were inevitably formed during high‐temperature fabrication steps [34, 57, 58]. These Ba‐rich phases, known to be electrochemically inactive [34], were removed after 10 min of alkaline leaching (Figure 3b). The removal of such particles is also attributed to the enhanced surface reactivity of alkaline‐leaching‐treated electrodes. Moreover, cross‐sectional SEM images (Figure S7) of the symmetrical cells confirm that the adhesion between the PBCC electrode and SDC electrolyte remains intact, with no evidence of delamination, cracking, or interfacial damage, indicating that the alkaline leaching treatment did not adversely affect interfacial integrity.

We also observed changes in surface crystallinity using HRTEM. The bare PBCC electrode exhibited high crystallinity (Figure 3c), with the calculated lattice constant (*a* = *b* = 3.86 Å) from interplanar spacing in agreement with X‐ray diffraction (XRD) results (Figure S8a) and previous reports [40, 43]. The magnified (110) and (102) peaks of XRD patterns in Figure S8b did not exhibit peak shifts after treatment, indicating that changes in the composition and structure of the electrode as a result of alkaline leaching only affect the topmost layers of its surface. After 10 min of alkaline leaching, an amorphous layer with a 2–4 nm thickness was observed on the PBCC electrode's surface. Moreover, this amorphous layer was maintained even after the operation at 550°C for 120 h (Figure S9).

The effect of amorphous surfaces on electrode performance has not yet been clearly elucidated. However, several studies have proposed mechanisms by which amorphous layers might influence electrochemical activity. For example, Choi et al. reported that the amorphous La_0.6_Sr_0.4_CoO_3‐δ_ demonstrated a higher oxygen p‐band center than its crystalline counterpart, indicating a lower oxygen removal energy of amorphous oxides [17]. Also, Cavallaro et al. suggested that amorphous La_0.8_Sr_0.2_CoO_3‐δ_ showed higher oxygen exchange kinetics and diffusivities compared to its crystalline state [47]. Based on such previous studies, it is plausible that the observed amorphous layer in our study contributed to the enhancement of electrode performance. The significant improvement in surface oxygen exchange kinetics, as discussed in Figure 2c,d, suggests that the amorphous layer may played a crucial role. The maintained amorphous layer over prolonged operation indicates that this modification is stable and beneficial. Further investigations are required to understand the underlying mechanisms fully, but our observations align with studies indicating that amorphous surfaces can improve electrode activity.

**FIGURE 4:**
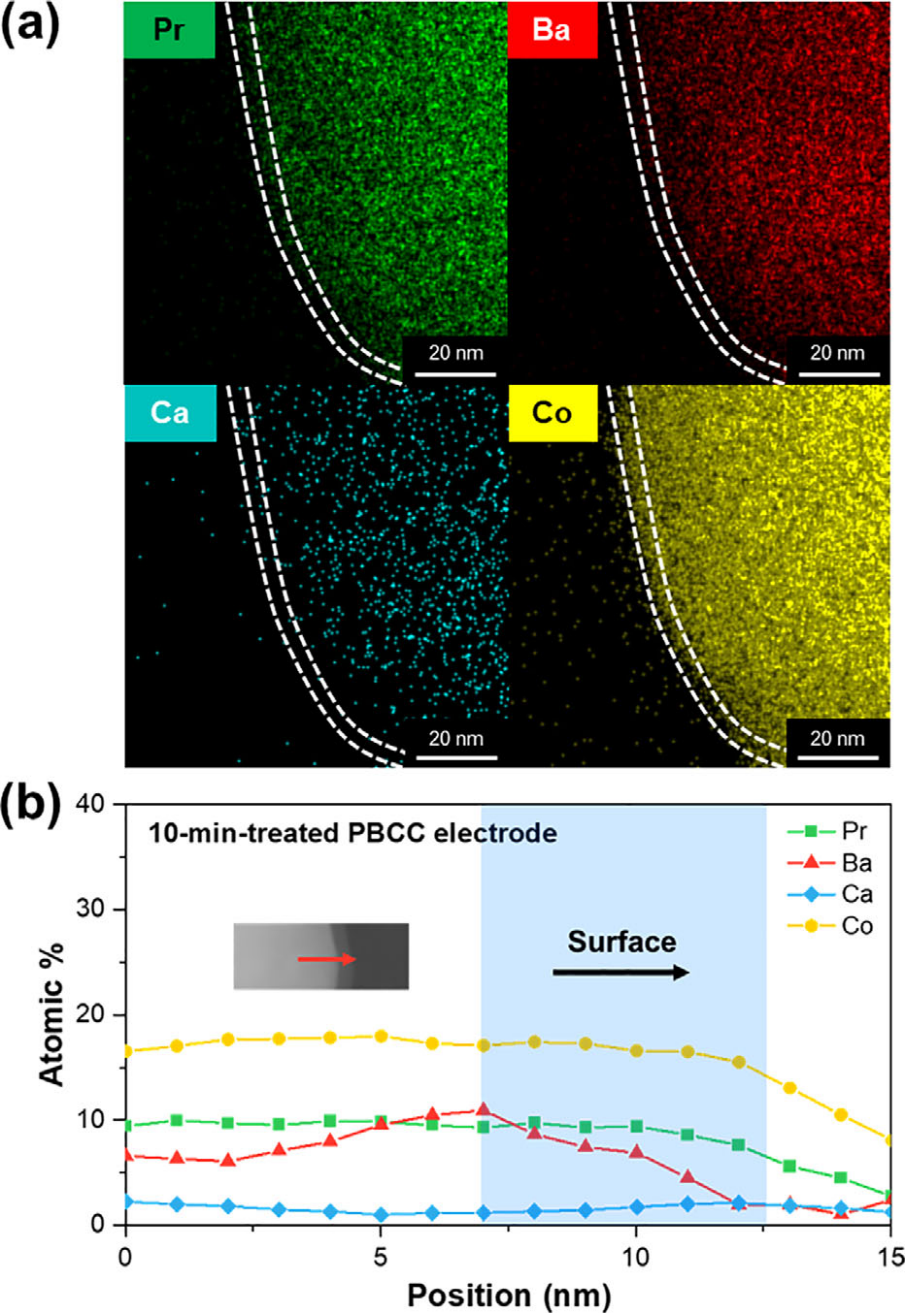
Changes in the surface composition of PrBa_0.8_Ca_0.2_Co_2_O_5+δ_ (PBCC) electrode after alkaline leaching. (a) Energy‐dispersive X‐ray spectroscopy mapping results of the 10 min‐treated PBCC electrode. The cobalt‐rich layer was observed on the surface of the PBCC electrode after alkaline leaching. (b) The line scan data along the arrow in the inset image.

Next, we investigated how the surface composition of PBCC changes upon the leaching process by observing TEM‐EDS mapping. Selective dissolution of barium and calcium led to the formation of a cobalt‐rich layer on the surface of the PBCC electrode (Figure 4a). The line‐scanned data, along with the red arrow, also confirmed the cobalt‐rich composition of the topmost layers (Figure 4b). The formation of a cobalt‐rich surface was further supported by inductively coupled plasma‐mass spectrometry (ICP‐MS) analysis conducted on the KOH solution used for alkaline leaching (Figure S10 and Table S2). The amount of barium and calcium cations leached into the KOH medium was significantly larger when the anodic bias was applied, while praseodymium and cobalt cations were rarely dissolved into the alkaline solution regardless of the applied bias. Platinum content was also analyzed to evaluate the possibility of surface contamination from the current collector during treatment. The results showed that the amount of dissolved Pt was negligible, confirming that it does not influence the observed surface composition or electrode properties. During the TEM observation, catalytically active cobalt oxide nanoparticles [18, 52, 53, 54] were also observed (Figure S11), which were formed from the cobalt (oxy)hydroxide layer as a result of alkaline leaching. This observation indicated that the applied anodic bias promotes the selective dissolution of alkaline earth metal cations, resulting in a cobalt‐rich PBCC surface. Along with the previous observation of reduced barium species and an amorphous layer, this cobalt‐rich surface was also depicted as one of the reasons for the enhanced ORR activity [34, 36, 37].

**FIGURE 5:**
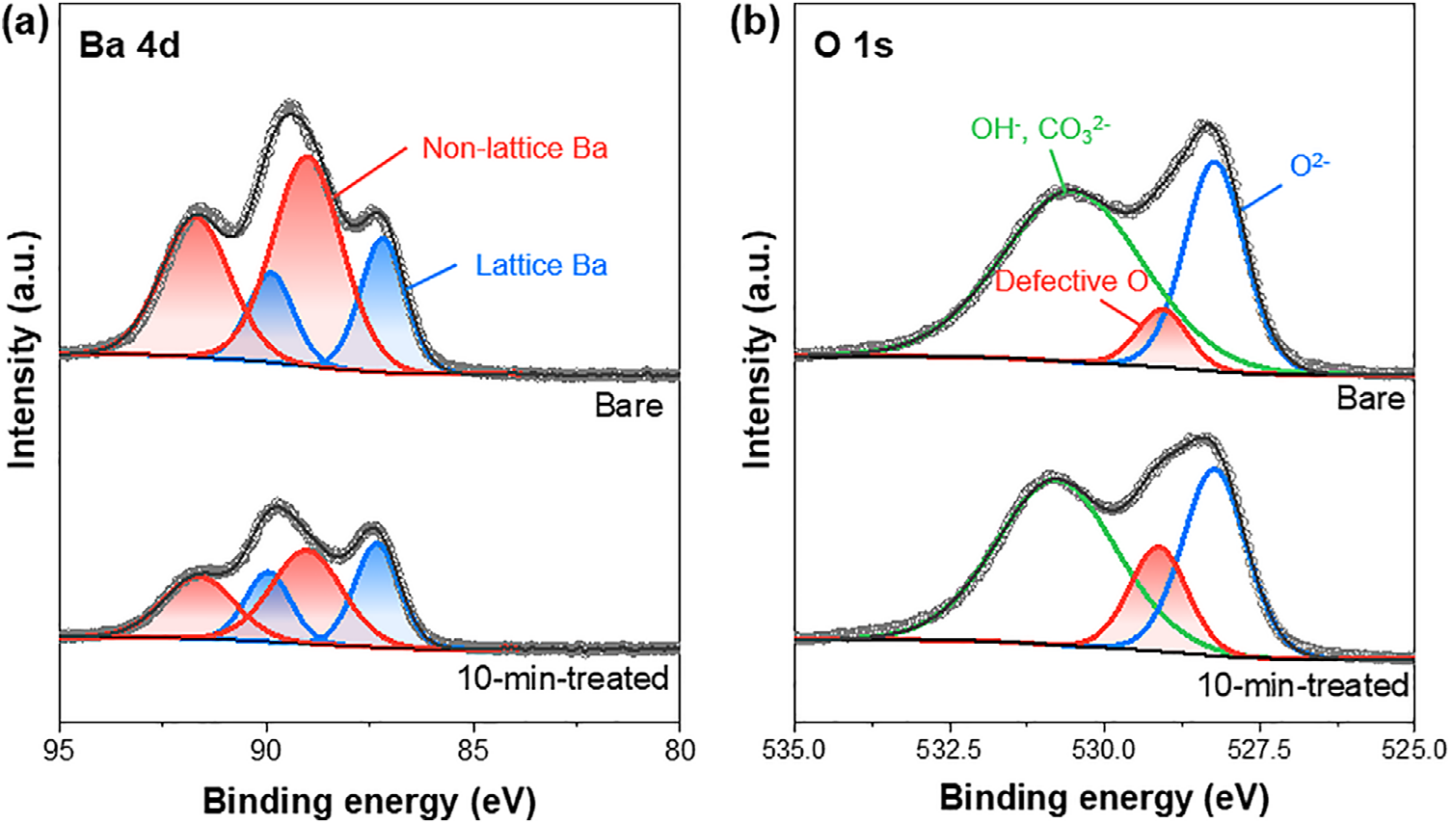
X‐ray photoelectron spectroscopy results of bare and 10 min‐treated PrBa_0.8_Ca_0.2_Co_2_O_5+δ_ electrodes. (a) Ba 4d spectra were deconvoluted by non‐lattice barium and lattice barium. (b) O 1s spectra were deconvoluted by O^2−^, defective oxygen, and non‐lattice oxygen, including OH^−^ and CO_3_^2−^.

Changes in the surface chemistry were further analyzed by XPS spectra to probe the origin of the improved ORR activity. The Ba 4d core‐level spectra (Figure 5a) were deconvoluted into two doublets. The red fitted lines were associated with the non‐lattice barium species at near 89.0 and 91.7 eV, while the blue fitted lines corresponded to lattice barium species at near 87.2 and 89.9 eV [59]. The non‐lattice barium species arose from the unintended surface cation segregation during the heat treatment process for powder synthesis and cell fabrication [57, 58]. The non‐lattice barium to lattice barium ratio (*I_non‐lattice Ba_*/*I_lacttice Ba_, I*: integrated area under the corresponding XPS peak) decreased from 2.35 to 1.40 after 10 min of alkaline leaching. This observed decrease in non‐lattice barium aligns with previous reports attributing this phenomenon to enhanced electrochemical activity [34]. This observation corroborates the findings from SEM images (Figure 3a,b), which reveal the removal of surface barium‐rich secondary phases upon alkaline leaching.

The O 1s XPS spectra (Figure 5b) were deconvoluted into three components, consistent with previous literature reports [60, 61, 62, 63]. The highest binding energy peaks (530.52 and 530.98 eV for bare and 10 min‐treated samples, respectively), denoted in green, were attributed to non‐lattice oxygen species, including OH^−^ and CO_3_^2−^. Conversely, the lowest binding energy peaks (528.23 and 528.43 eV for bare and 10 min‐treated samples, respectively), depicted in blue, correspond to O^2−^ species. The red‐colored peaks (529.07 and 529.33 eV for bare and 10 min‐treated samples, respectively) were assigned to defective oxygen species, which include O^−^, O_2_^2−^, and oxygen vacancies [60, 64, 65]. A notable increase in the ratio of defective oxygen to lattice oxygen was observed from 0.229 to 0.501 upon alkaline leaching. This enhancement in the relative abundance of defective oxygen species, known to be highly active for the ORR [63], is consistent with the observed improvement in electrochemical activity.

Moreover, the Ba‐deficient and Co‐rich surface was also confirmed by the qualitative analysis of the XPS spectra. The surface mole fraction of each element was calculated using the following equation:

(2) $$X_{i}=\frac{I_{i}/S_{i}}{\sum_{j}I_{j}/S_{j}}$$

where X_i_ is the mole fraction, I_i_ is the integrated XPS spectrum area, and S_i_ is the relative sensitivity factor of element i. To avoid peak overlap and ensure higher accuracy, Ba 3d and Co 2p were not used despite being major peaks. Instead, Ba 4d, Pr 3d, Ca 2p, and Co 3p core‐level spectra were selected for quantitative calculation. The resulting surface composition was demonstrated in Figure S12a. After alkaline leaching, the Ba and Ca ratios significantly decreased, whereas the Co ratio was relatively increased. When directly comparing Ba and Co (Figure S12b), Ba is clearly reduced while Co is relatively enriched. These results further support the formation of a Ba‐deficient surface layer following alkaline leaching.

The reduction in non‐lattice barium species upon alkaline leaching suggests a decrease in segregated surface cations, which can hinder active sites. The alignment of this observation with improved electrochemical activity supports the notion that removing surface barium‐rich secondary phases enhances ORR performance. Similarly, the significant increase in defective oxygen species observed in O 1s spectra may serve as more active sites. Therefore, the increase in the ratio of defective oxygen to lattice oxygen correlates with the enhanced ORR activity after alkaline leaching treatment. These findings are consistent with previous studies reporting that reducing surface cation segregation and increasing oxygen defect concentration positively affect electrochemical performance.

To further exclude the possibility that the observed improvements originated from unintended elemental contamination, including platinum from the current collector and potassium from the alkaline medium, XPS survey spectra were analyzed (Figure S13a). Only signals corresponding to the intrinsic elements of the PBCC electrode were observed, along with carbon from surface‐absorbed species by the ambient air. No Pt 4f signal was detected in the 70–80 eV region (Figure S13b), and K 2p peaks were also absent in the 294–297 eV range (Figure S13c). These results confirm that neither platinum redeposition nor potassium contamination occurred during the alkaline leaching treatment. Therefore, the observed enhancements in ORR activity can be attributed to the intrinsic surface chemical modifications induced by the leaching process, rather than any extrinsic elemental contribution.

To demonstrate the practical relevance of our strategy, alkaline leaching was applied to the PBCC air electrode of the single cell, and its electrochemical performance was evaluated. Figure S14 presents the fabricated single cell, in which a screen‐printed Ag grid was used for current collection, exerting a negligible influence on electrochemical performance. The cross‐sectional SEM image of the fabricated single cell configuration with Ni‐(Y_2_O_3_)_0.08_(ZrO_2_)_0.92_ (YSZ)|YSZ|SDC|PBCC is shown in Figure S15. The cell architecture remained largely unchanged after alkaline leaching treatment (Figure S16).

**FIGURE 6:**
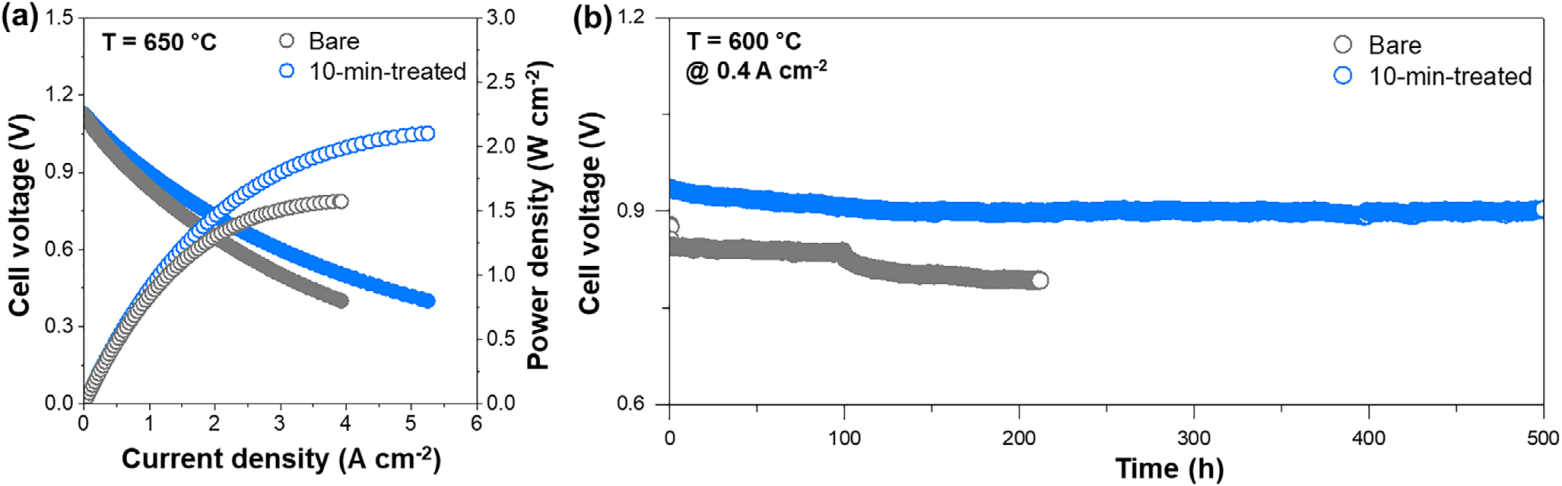
(a) *I–V–P* curves and (b) 500 h long‐term stability test results of the bare and 10 min‐treated single cell with the configuration of Ni‐(Y_2_O_3_)_0.08_(ZrO_2_)_0.92_‐ (YSZ)|YSZ|Sm_0.2_Ce_0.8_O_2‐δ_|PrBa_0.8_Ca_0.2_Co_2_O_5+δ_.

At 650°C, the maximum power density (MPD) increased from 1.58 to 2.10 W cm^−2^, a 33% enhancement (Figure 6a). A comparable improvement was obtained at 600°C, where the MPD enhanced from 0.79 to 1.01 W cm^−2^, corresponding to a 28% increase (Figure S17). Moreover, the MPD reached 3.21 W cm^−2^ at 700°C after 10 min of alkaline leaching (Figure S18). The alkaline‐leaching‐treated single cell's performance is comparable to the previously reported record‐level Ni‐YSZ‐based single cells, as demonstrated in Figure S19.

The impedance spectra revealed that the overall decrease in polarization resistance matched the reduction in air electrode resistance, while the ohmic resistance remained unchanged, as shown in EIS spectra measured at OCV and under biases of 0.9 and 0.8 V (Figure S20a,c). The slopes of *I–V* curves at 0.9 and 0.8 V were consistent with the area‐specific resistance of the single cells (Figure S20b,d). Furthermore, DRT analyses of the EIS spectra confirmed the selective effect of alkaline leaching on the air electrode, as the peaks in the intermediate frequency region decreased significantly (Figure S21). Therefore, along with the previously demonstrated chemical stability of Ni‐YSZ in alkaline solution [66, 67], the cell performance enhancement can be primarily attributed to the surface activation of the air electrode by alkaline leaching.

We additionally conducted long‐term stability tests on single cells to reliably assess their durability under operating conditions. A 500 h test was carried out on the treated cell, while the bare cell was tested for 200 h. As shown in Figure 6b, the alkaline‐leaching‐treated cell maintained stable performance throughout the 500 h operation, with a degradation rate measured to be 0.07 mV h^−1^. Consistently, EIS analysis of the 10 min‐treated single cell (Figure S22) revealed that the polarization resistance increased only slightly from 0.51 to 0.53 Ω cm^2^ after 200 h at 600°C, corresponding to only 3.9% degradation. These results underscore the potential of the alkaline‐leaching strategy for enabling long‐term and stable operation in practical SOFC applications.

Alkaline leaching has significant technical implications, as an obvious improvement in the activity of the PBCC air electrode was accomplished. Building on this finding, our strategy enables targeted engineering of the surface composition and structure of the SOFC cells before stacking. The cell manufacturing processes, which have been established considering various mechanical factors, such as thermal expansion coefficients, need not be changed. Notably, other compartments in SOFCs still can be utilized, considering the stability of commercialized electrolyte materials (e.g., YSZ) and fuel electrode materials (e.g., Ni‐YSZ composite) in alkaline solution [66, 67], as verified through single cell tests. Also, given previous studies indicating surface reconstruction phenomena during alkaline water splitting reactions involving transition metal cations (e.g., Ni and Fe) [25, 68, 69], our approach holds promise for extending to other mixed‐conducting oxide materials. Moreover, this technique offers an increase in the number of active sites without increasing the usage of critical elements, i.e., cobalt. Considering the wide applicability of perovskite oxides, alkaline leaching presents an economically feasible surface treatment method to enhance the performance of electrochemical devices, including solid oxide electrolysis cells, protonic ceramic fuel cells [70], and metal‐air batteries [71].

## 3 Conclusions

In this pioneering application of alkaline leaching to SOFC air electrodes, the electrochemical performance of PBCC electrodes was significantly enhanced through the selective dissolution of inert species and the exposure of catalytically active species. A remarkable improvement in ORR activity was recorded when anodic bias was applied to the PBCC electrode using a simple and conventional 3‐electrode system with an alkaline solution. After just 10 min of alkaline leaching, the PBCC electrode exhibited an electrode resistance of only 0.019 Ω cm^2^ at 650°C. Furthermore, a 7.5 fold increase in ORR activity relative to the untreated PBCC electrode was observed at 450°C. Additionally, single‐cell measurements revealed a 33 % increase in maximum power density at 650°C after alkaline leaching, recording 2.10 W cm^−2^. This enhanced electrochemical reactivity demonstrates considerable potential for reducing the operating temperatures of SOFCs.

Comprehensive analyses were conducted to examine the surface modifications of the PBCC electrode and elucidate the mechanisms underlying the enhanced electrochemical performance resulting from alkaline leaching. Our findings indicate that, following alkaline leaching, three key alterations, previously recognized as advantageous for ORR, occurred on the PBCC electrode surface. First, the surface of the PBCC electrode transitioned to an amorphous state. Second, the surface composition became enriched in cobalt. Finally, surface chemistry was modified, with a reduction in inactive barium species and an increase in active oxygen species.

In summary, we have presented alkaline leaching as an innovative and promising strategy for enhancing the surface electrochemical reactivity of PBCC air electrodes for SOFCs. This approach, requiring only a 10 min treatment at room temperature and ambient pressure, provides a straightforward method for surface modification. The applicability of alkaline leaching extends beyond SOFC electrodes, demonstrating versatility across perovskite oxides in diverse structures such as powders, thin films, and porous electrodes. Considering the manifold advantages of perovskite‐oxide‐based materials, alkaline leaching emerges as a novel surface modification strategy, prompting further exploration for the development of high‐performance (electro)chemical catalysts.

## 4 Experimental Procedures

### 4.1 Fabrication of PBCC Symmetrical Cells

To test the ORR activity of the PBCC electrodes, symmetrical cells with the PBCC|SDC|PBCC configuration were fabricated. Commercial SDC powder (Fuelcellmaterials, Inc.) was uniaxially pressed and sintered at 1450°C for 5 h to prepare the dense SDC electrolyte pellets, as introduced in our previous report [72]. Commercial PBCC powder (KCeracell Co., Ltd.) was weighed and mixed with ink vehicle (Fuelcellmaterials, Inc.) at a 1:1 weight ratio. The mixture was then ball‐milled with zirconia balls in ethanol (99%) for 24 h. The excess ethanol was dried in an oven at 80°C to achieve sufficient viscosity. The prepared PBCC ink was screen‐printed on both sides of the SDC electrolyte and sintered at 950°C for 2 h to fabricate the desired interconnected and porous PBCC electrodes.

### 4.2 Alkaline Leaching of PBCC Electrodes

For alkaline leaching, a conventional 3‐electrode system with 1 m KOH electrolyte was utilized. A graphite rod and Hg/HgO/1 m KOH electrode were used as the counter and reference electrodes, respectively. A PBCC symmetrical cell was loaded with a Pt clip so the PBCC electrode could serve as the working electrode. At a constant voltage mode, 1 V was applied between the working and reference electrodes by a potentiostat (BioLogic, VMP‐300) to induce alkaline OER, corresponding to the current density of approximately 35.7 mA cm^−2^. Both sides of the PBCC symmetrical cell were treated under the same conditions. After alkaline leaching, the cell was thoroughly rinsed with deionized water to remove the remaining KOH solution on the surface and dried with N_2_ (99.999 %).

### 4.3 Fabrication of Single Cells

Fuel‐electrode‐supported cells with the configuration of Ni‐YSZ|YSZ|SDC|PBCC were fabricated [73]. The Ni‐YSZ fuel electrode and YSZ electrolyte were fabricated by tape casting and co‐sintering. To prepare the slurries for both the fuel electrode support and the fuel electrode functional layer, a mixture consisting of 60 wt.% NiO (Fuelcellmaterials, Inc.) and 40 wt.% YSZ (Fuelcellmaterials, Inc.) was mixed with solvents, a binder, and starch as a pore former, followed by ball‐milling for 48 h. The YSZ electrolyte layer was prepared in the same manner, without the use of a pore former. The resulting green sheets were laminated by warm isostatic pressing at 70°C under a pressure of 20 MPa for 15 min to regulate the thickness. The laminated sheets were then sintered at 1400°C for 5 h. The SDC buffer layer was subsequently fabricated by ultrasonic spray coating onto the electrolyte layer and sintered at 1300°C for 3 h. The PBCC air electrode was screen‐printed onto the buffer layer, followed by sintering at 950°C for 2 h. Alkaline leaching was applied to the PBCC air electrode.

### 4.4 Electrochemical Characterization

EIS measurements were conducted to evaluate the electrochemical activity of bare and alkaline‐leaching‐treated PBCC symmetrical cells. The cells were loaded using custom‐made Pt clips, placed in a tube furnace, and impedance spectra were measured at the temperature range of 450°C–650°C and frequency range of 10 mHz—2 MHz with a sinus amplitude of 40 mV. The *pO_2_*‐dependency of the spectra was examined under various oxygen partial pressures with the range of 0.01–1 atm at 600°C. For ease of interpretation, distribution of relaxation times analyses was conducted with open‐source DRTtools software [74, 75]. The EIS data were used to obtain the compartment of electrode resistance with respect to the frequency.

Electrochemical performance of single cells was also examined for both untreated and alkaline‐leaching‐treated samples. Ag current collectors were formed by screen‐printing in a grid on both the fuel and air electrode surfaces. The cells were mounted on an alumina tube and sealed with a ceramic adhesive (Aremco). Electrical connections were established using a four‐probe setup with Pt mesh and Pt lead wires. Humidified hydrogen (3 % H_2_O) was supplied to the fuel electrode, while ambient air was delivered to the air electrode at a constant flow rate of 100 mL min^−1^, regulated by a digital mass flow controller. Electrochemical performance was evaluated at 650°C–600°C using a potentiostat. During the single cell operation, EIS measurements were conducted over a frequency range of 0.1 Hz—1 MHz with a sinus amplitude of 20 mV.

### 4.5 Physicochemical Characterizations

The crystal structures of the bare and alkaline‐leached PBCC electrodes were investigated by XRD (Rigaku Ultima IV) with the 2theta range of 20°–60°, a scan rate of 5° min^−1^, and a step size of 0.01°. The porous structures of the PBCC electrodes were observed by SEM (Hitachi SU8230). For XRD and SEM, PBCC electrodes were fabricated on (0001) single‐crystal Al_2_O_3_ substrates and analyzed before and after alkaline leaching.

TEM (Talos F200X) was used to probe the surface crystallinity change. The porous PBCC electrodes were scraped with a razor blade and sonicated in ethanol for even dispersion. Then the sample was prepared on the holey‐carbon‐coated copper grid and dried at 60°C for 12 h. To obtain high‐resolution images, 200 kV of acceleration voltage was applied. Also, the samples were line‐scanned with EDS to characterize the surface composition change after alkaline leaching. For a more in‐depth analysis of surface chemistry change after alkaline leaching, XPS (Thermo Scientific Nexsa G2) analysis and ICP‐MS (Agilent ICP‐MS 7700S) were conducted.

## Author Contributions

W.J. and T.H.S. conceived and supervised this work. Y.H. and H.K. fabricated materials and performed the following experiments: alkaline leaching, electrochemical testing, XRD, SEM, TEM, and XPS analyses. Y.H. conducted DRT and ICP‐MS analyses. H.K. invented the concept. S.W.L. and S.R. fabricated single cells and performed electrochemical testing. Y.B.K. assisted in establishing the conditions for alkaline leaching. S.J. assisted in DRT analysis. S.K., J.L., and H.S. assisted in constructing the 3‐electrode system. S.N. fabricated PBCC ink. Y.H., H.K., S.W.L., T.H.S., and W.J. contributed to the manuscript writing.

## Conflicts of Interest

There are no conflicts of interest to declare.

## Supporting information




**Supporting File**: adma72368‐sup‐0001‐SuppMat.docx.

## Data Availability

The data that support the findings of this study are available in the supplementary material of this article.
