# Supplementary material for: Alkaline Leaching: A Facile Surface Activation Strategy to Improve the Reactivity of Air Electrodes for Solid Oxide Fuel Cells
Source: Adv Mater. 2026 Jan 30;38(33):e11053. doi: 10.1002/adma.202511053 (PMC13261383; doi:10.1002/adma.202511053)
Supplement: Supplementary file 1 — Supporting File: adma72368‐sup‐0001‐SuppMat.docx. [file ADMA-38-e11053-s001.docx]

**Supporting Information**

**Alkaline Leaching: a Facile Surface Activation Strategy to Improve the Reactivity of**

**Air Electrodes for Solid Oxide Fuel Cells**

Yeongtaek Hong,^1+^ Hyunseung Kim,^2a+^ Sang Won Lee,^3+^ Yong Beom Kim,^2^ SungHyun Jeon,^2b^ Sangwoo Kim,^4c^ Hainan Sun,^5^ Jeongah Lee,^1^ Seongwoo Nam,^2^ Seungwoo Roh,^1^ Tae Ho Shin,^3*^ and WooChul Jung^1,2*^

^1^ Department of Materials Science and Engineering, Seoul National University (SNU), Seoul, Republic of Korea

^2^ Research Institute of Advanced Materials (RIAM), Seoul National University (SNU), Seoul, Republic of Korea

^3^ Korea Institute of Ceramic Engineering and Technology (KICET), Jinju, Republic of Korea

^4^ Department of Materials Science and Engineering, Korea Advanced Institute of Science and Technology (KAIST), Daejeon, Republic of Korea

^5^ School of Chemistry and Chemical Engineering, Nantong University, Nantong, Jiangsu, P. R. China

^a^ Current Address: Energy Research Institute @ NTU (ERI@N), Interdisciplinary Graduate School, Nanyang Technological University, Singapore

^b^ Current Address: Department of Materials Science and Engineering, Northwestern University, Evanston, Illinois, USA

^c^ Current Address: Platform Technology Research Center, LG Chem, Seoul, Republic of Korea

^+^ These authors contributed equally to this work.

* Correspondence: T. H. Shin (ths@kicet.re.kr) and W. Jung (wcjung@snu.ac.kr)

**Supporting Figures**


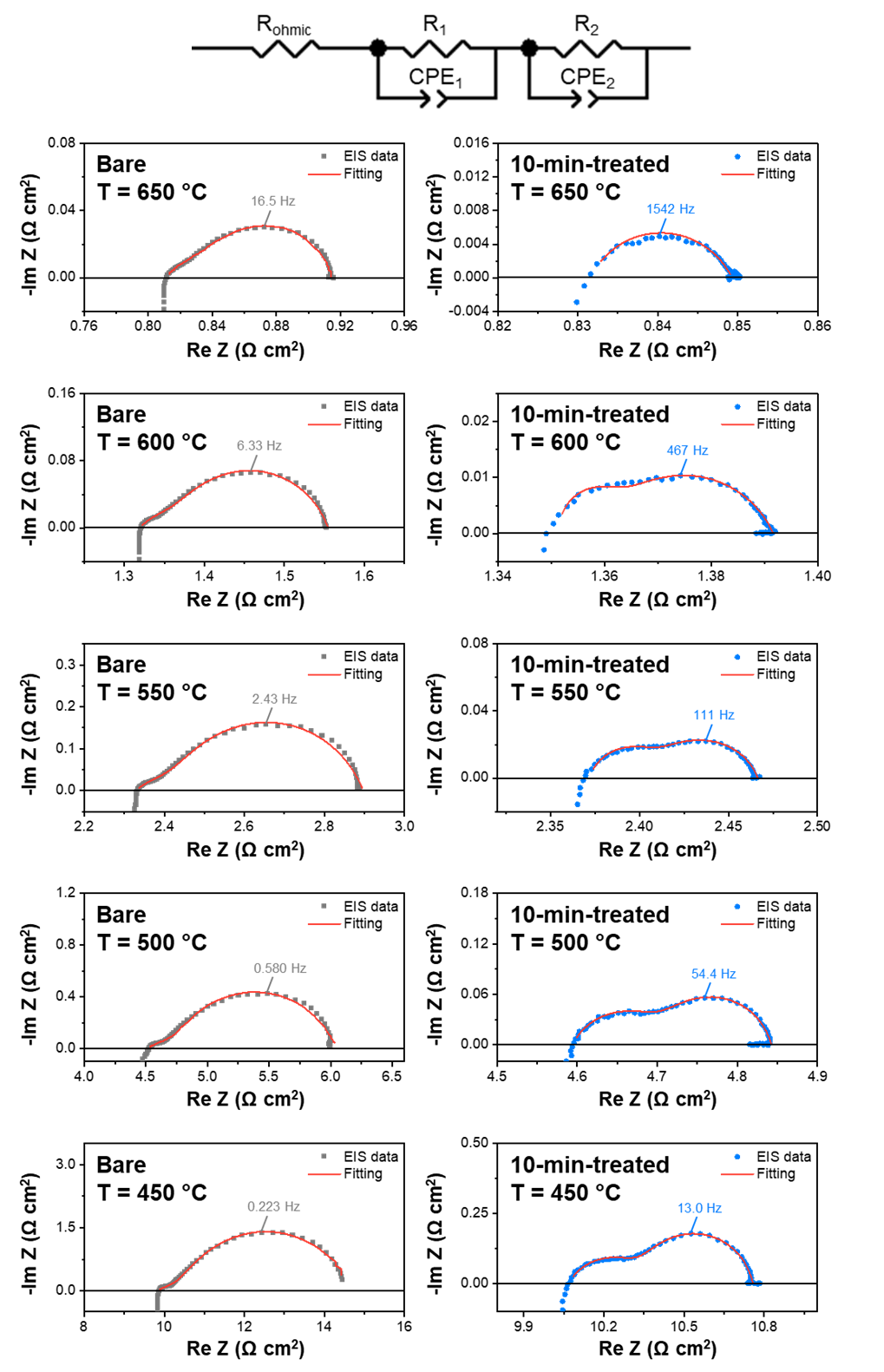


Figure S1. Equivalent circuit model and fitting results from the electrochemical impedance spectroscopy data of bare and 10-min-treated PrBa_0.8_Ca_0.2_Co_2_O_5+δ_ symmetrical cells with the configuration of Sm_0.2_Ce_0.8_O_2-δ_ electrolyte at 450 – 650 °C and *pO_2_* = 0.21 atm.

Table S1. Fitted resistance values extracted from impedance spectra of bare and 10-min-treated PrBa_0.8_Ca_0.2_Co_2_O_5+δ_ symmetrical cells with Sm_0.2_Ce_0.8_O_2-δ_ electrolyte.

| **Sample** | **T**  **(°C)** | **R_ohmic_**  **(Ω cm^2^)** | **R_1_**  **(Ω cm^2^)** | **R_2_**  **(Ω cm^2^)** | **R_electrode_**  **(Ω cm^2^)** |
| --- | --- | --- | --- | --- | --- |
| Bare | 650 | 0.814 | 0.072 | 0.031 | 0.10 |
|  | 600 | 1.32 | 0.18 | 0.058 | 0.24 |
|  | 550 | 2.33 | 0.45 | 0.12 | 0.57 |
|  | 500 | 4.51 | 1.2 | 0.30 | 1.5 |
|  | 450 | 9.83 | 4.0 | 0.85 | 4.9 |
| 10-min-treated | 650 | 0.828 | 0.0032 | 0.016 | 0.019 |
|  | 600 | 1.35 | 0.0087 | 0.032 | 0.041 |
|  | 550 | 2.37 | 0.036 | 0.058 | 0.094 |
|  | 500 | 4.59 | 0.12 | 0.13 | 0.25 |
|  | 450 | 10.1 | 0.29 | 0.40 | 0.69 |

Figure S2. An enhancement factor of alkaline-leaching-treated electrode resistance of PrBa_0.8_Ca_0.2_Co_2_O_5+δ_ electrodes at 450 – 650 °C. The electrode reactivity (inverse of the electrode resistance) of the sample after alkaline leaching was divided by that of the bare sample.


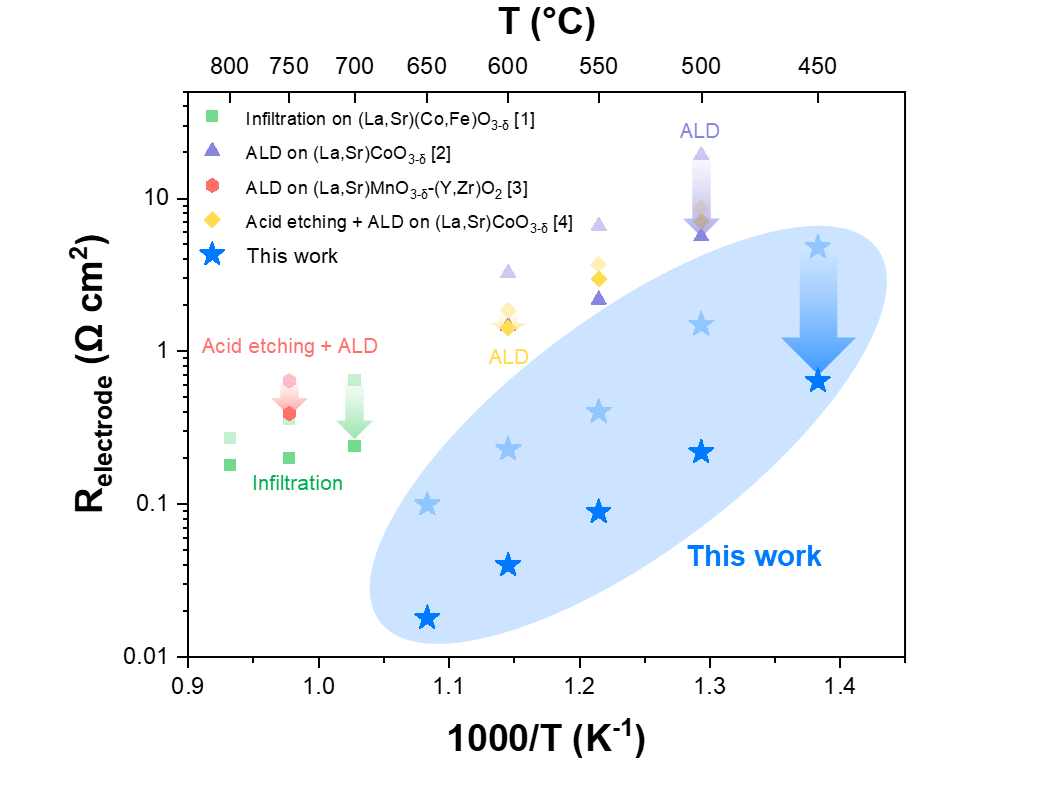


Figure S3. Arrhenius plots for comparing enhancement factors by previously reported surface modification strategies^[1–4]^ and this work.


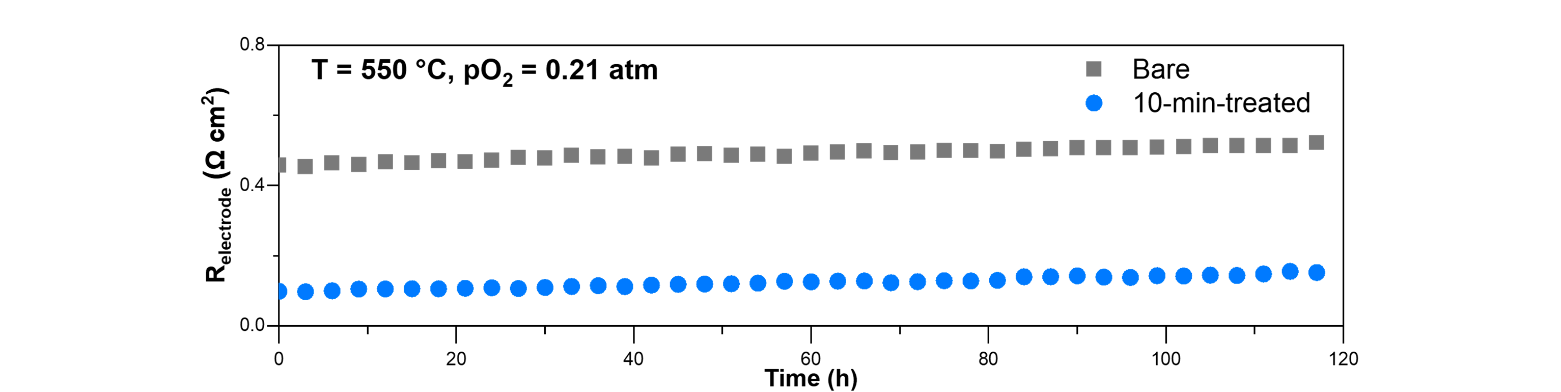


Figure S4. The 120-hour stability test results of the bare and 10-min-treated PrBa_0.8_Ca_0.2_Co_2_O_5+δ_ (PBCC) electrodes measured at 550 °C for 120 h with the symmetrical cell configuration of PBCC|Sm_0.2_Ce_0.8_O_2-δ_|PBCC.

Figure S5. Distribution of relaxation time plot of alkaline-leaching-treated PrBa_0.8_Ca_0.2_Co_2_O_5+δ_ electrode at *pO_2_* = 0.21 atm with a temperature range of 450 – 650 °C.


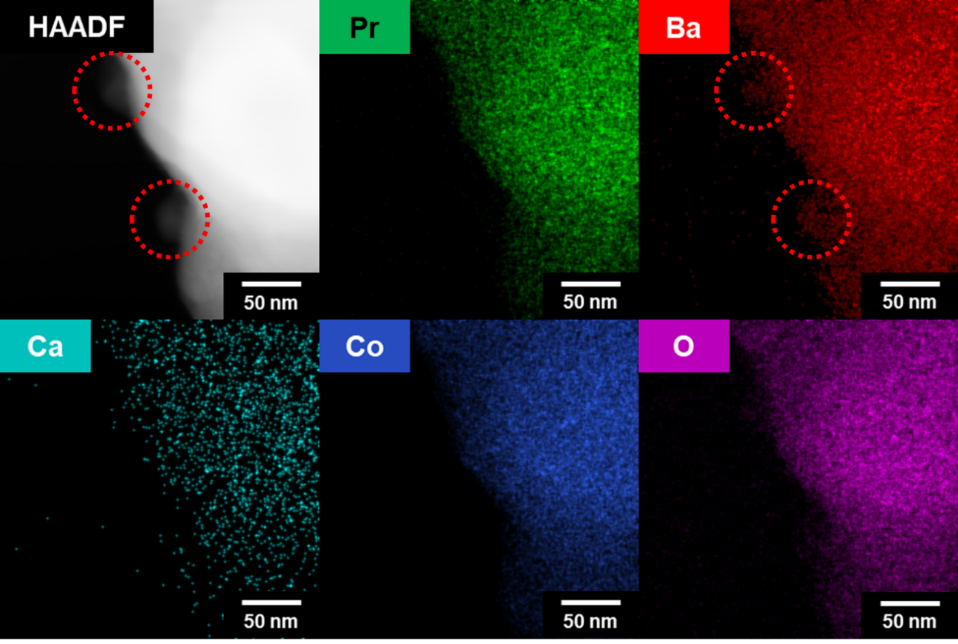


Figure S6. Energy dispersive X-ray spectroscopy results of the bare PrBa_0.8_Ca_0.2_Co_2_O_5+δ_ electrodes. BaO_x_ secondary phase particles are indicated as red circles.


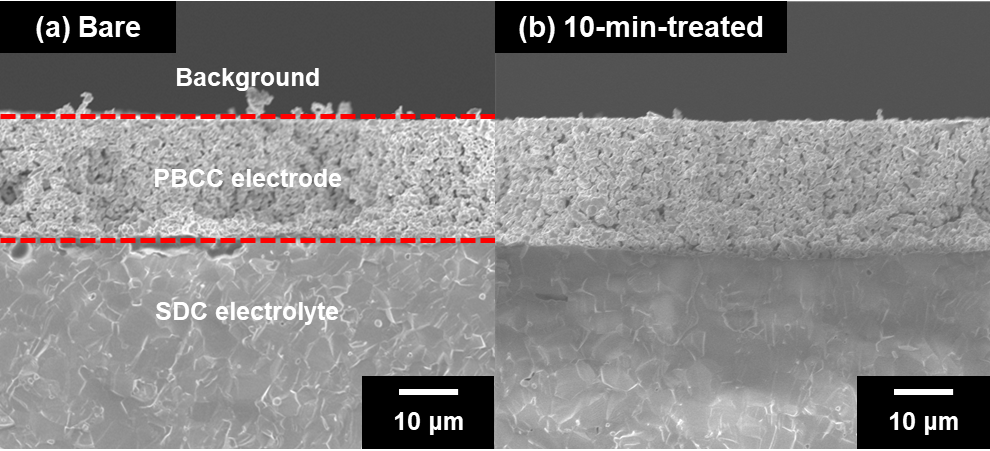


Figure S7. Cross-sectional scanning electron microscopy images of (a) bare and (b) 10-min-treated PrBa_0.8_Ca_0.2_Co_2_O_5+δ_ (PBCC)|Sm_0.2_Ce_0.8_O_2-δ_ (SDC)|PBCC symmetrical cells.


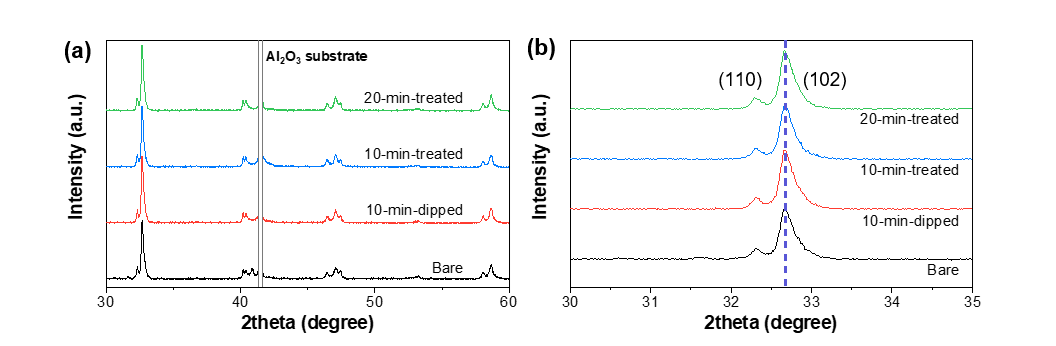


Figure S8. (a) X-ray diffraction patterns of bare and alkaline-leaching-treated PrBa_0.8_Ca_0.2_Co_2_O_5+δ_ electrodes fabricated on (0001) single-crystalline Al_2_O_3_ substrates and (b) the magnified (110) and (102) peaks, showing no significant shifts.


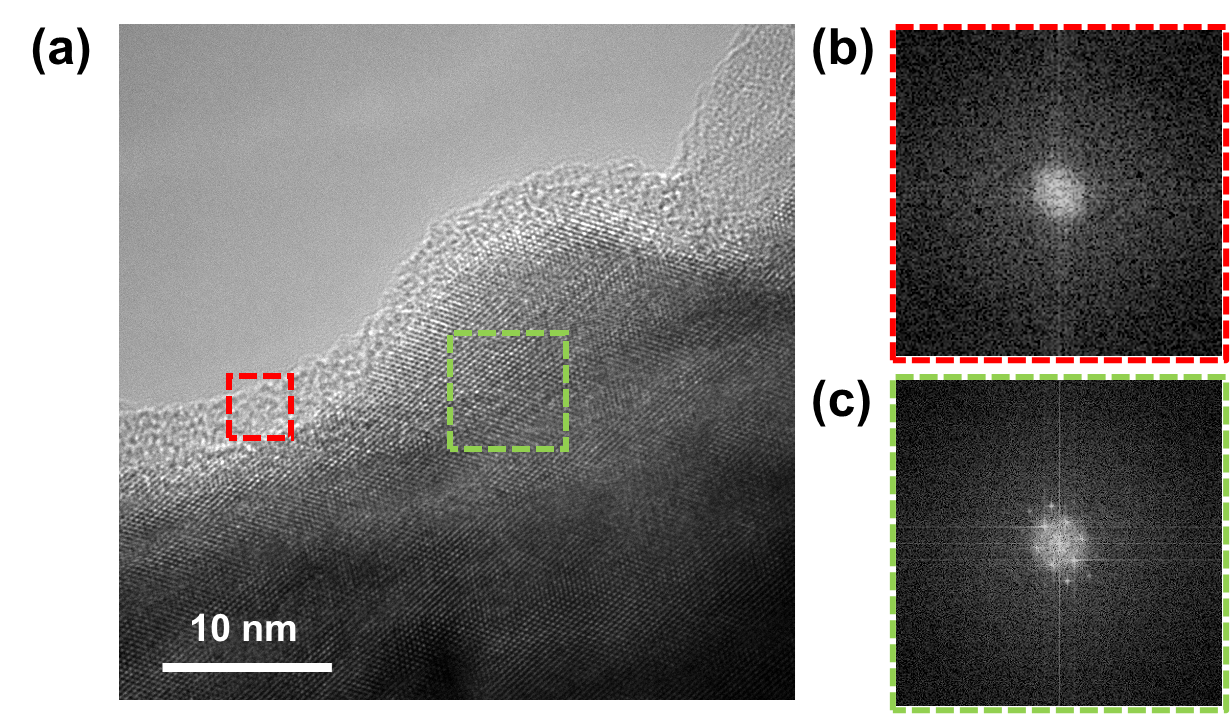


Figure S9. (a) High-resolution transmission electron microscopy images of 10-min-treated PrBa_0.8_Ca_0.2_Co_2_O_5+δ_ (PBCC) electrode after long-term electrochemical impedance spectroscopy measurement at 550 °C for 120 h. Fast Fourier transform patterns indicate the amorphous layer on the surface of the PBCC electrode was maintained (b) compared to the bulk (c).


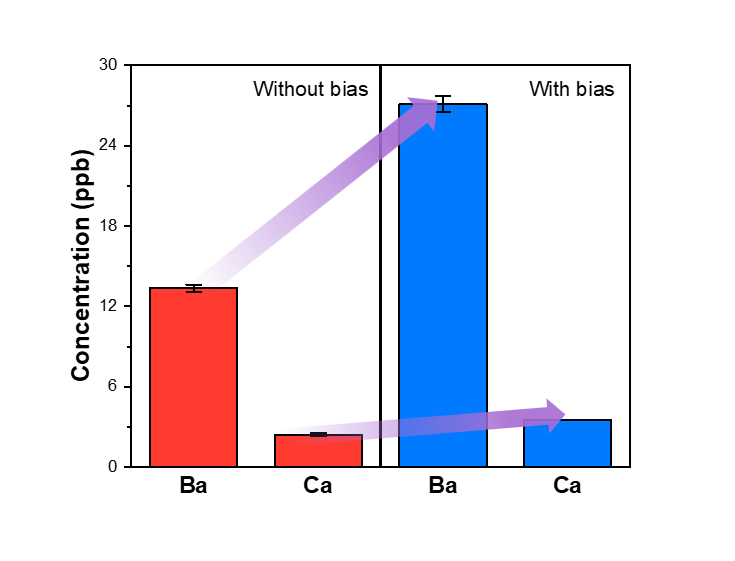


Figure S10. Inductively coupled plasma-mass spectrometry results of barium and calcium taken on the KOH solutions where the PrBa_0.8_Ca_0.2_Co_2_O_5+δ_ electrodes were just dipped without bias on the left and the sample was leached with bias on the right.

Table S2. Inductively coupled plasma-mass spectrometry results of praseodymium, barium, calcium, cobalt, and platinum taken on the KOH solutions, where the PrBa_0.8_Ca_0.2_Co_2_O_5+δ_ electrodes underwent the alkaline leaching treatment.

| Element | Concentration (ppb) |
| --- | --- |
| Pr | <0.001 |
| Ba | 27.10 |
| Ca | 3.52 |
| Co | <0.001 |
| Pt | 0.010 |


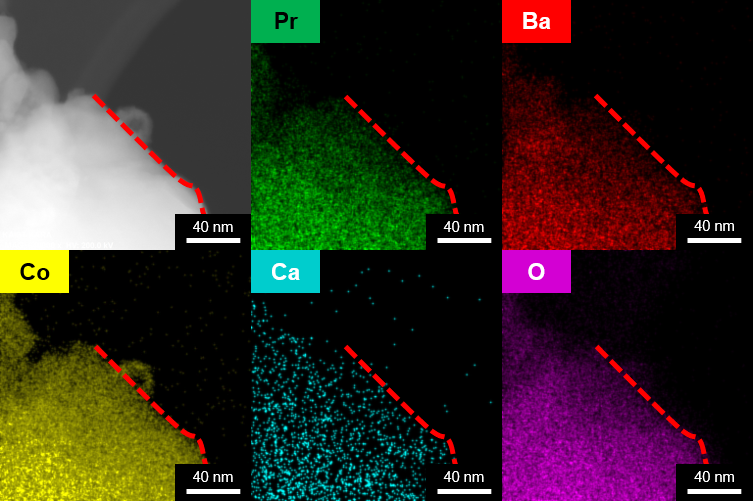


**Figure S11.** Transmission electron microscopy-energy-dispersive X-ray spectroscopy results of alkaline-leaching-treated PrBa_0.8_Ca_0.2_Co_2_O_5+δ_ electrode after annealing at 650 °C for 40 min, corresponding to the operating condition of solid oxide fuel cells.


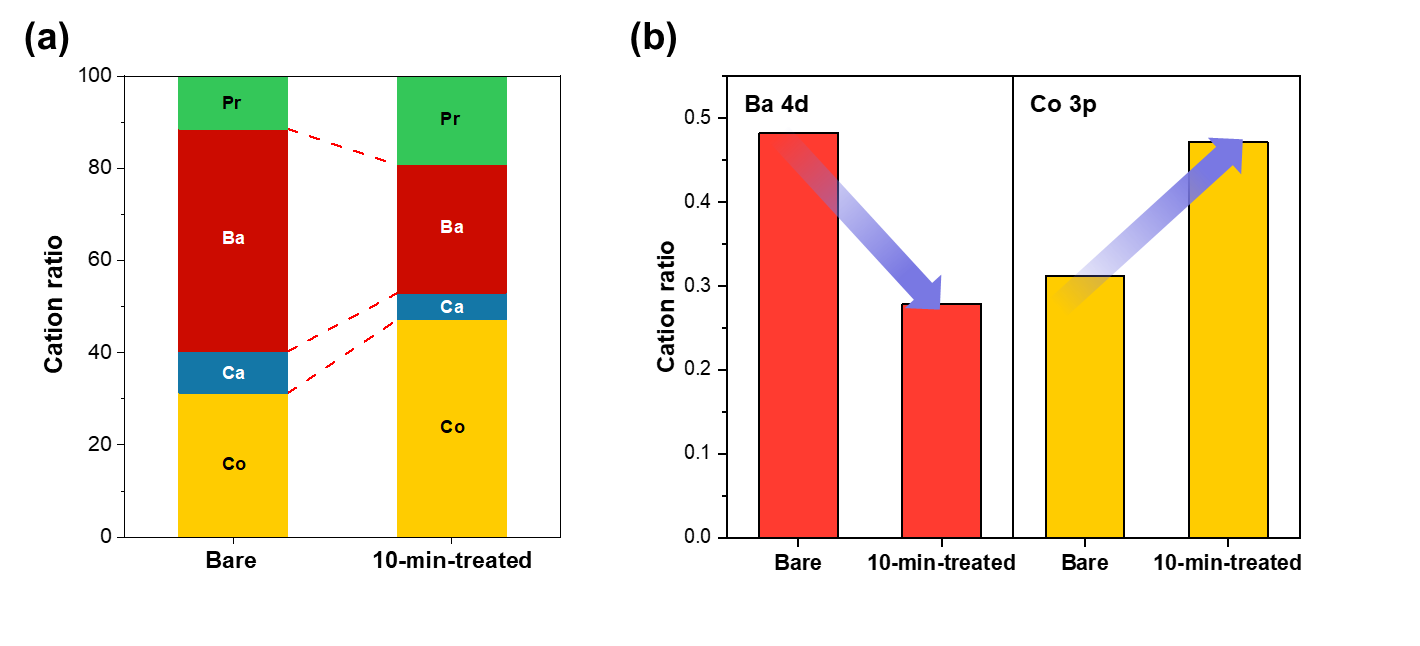


Figure S12. (a) Relative cation ratio calculated from X-ray photoelectron spectroscopy results of bare and 10-min-treated PrBa_0.8_Ca_0.2_Co_2_O_5+δ_ electrode. (b) Quantitative calculation results of Ba 4d and Co 3p.


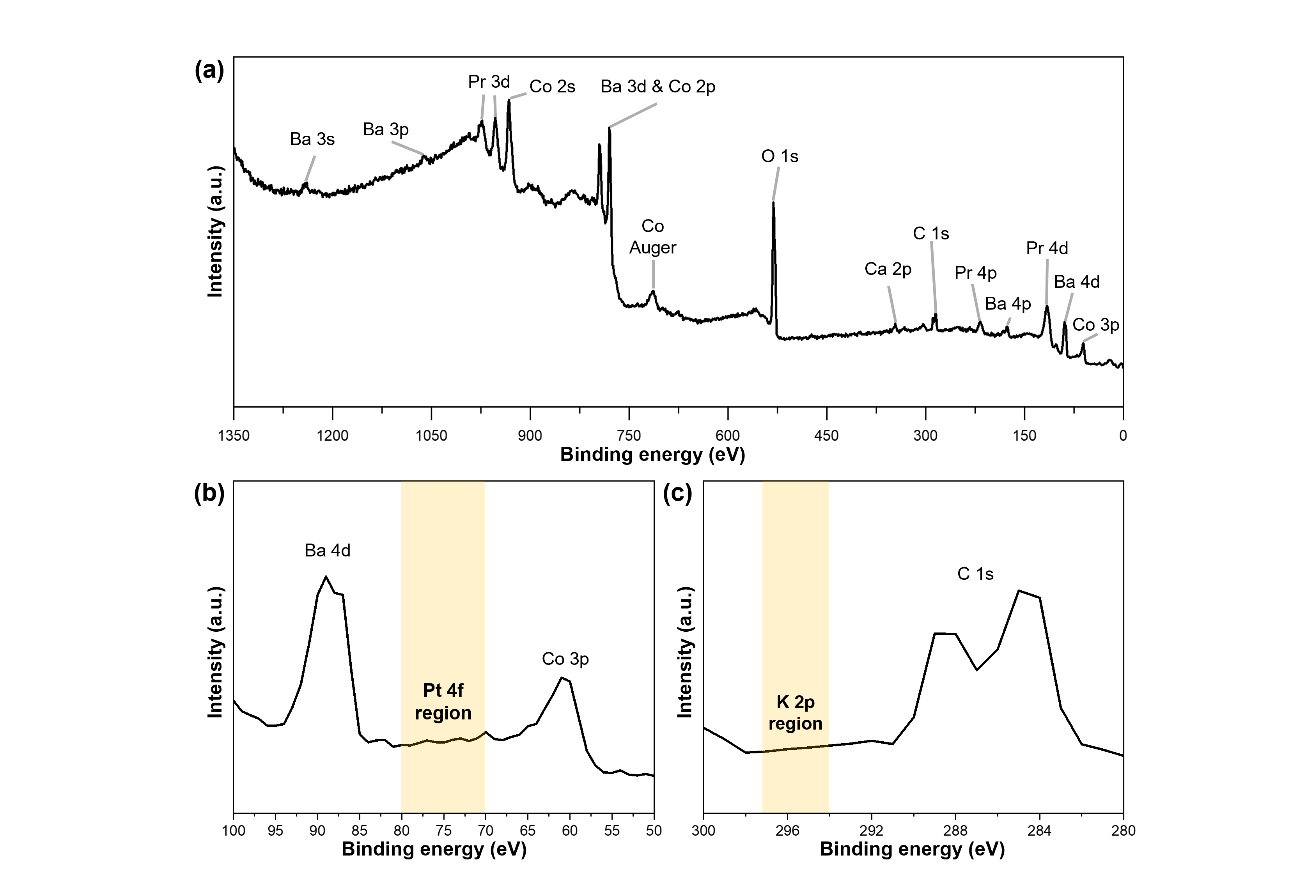


Figure S13. (a) X-ray photoelectron spectroscopy survey spectrum of 10-min-treated PrBa_0.8_Ca_0.2_Co_2_O_5+δ_ (PBCC) electrode. No additional element was detected. The focused (b) 80 – 100 eV region, including Pt 4f, and (c) 280 – 300 eV region, including K 2p.

**
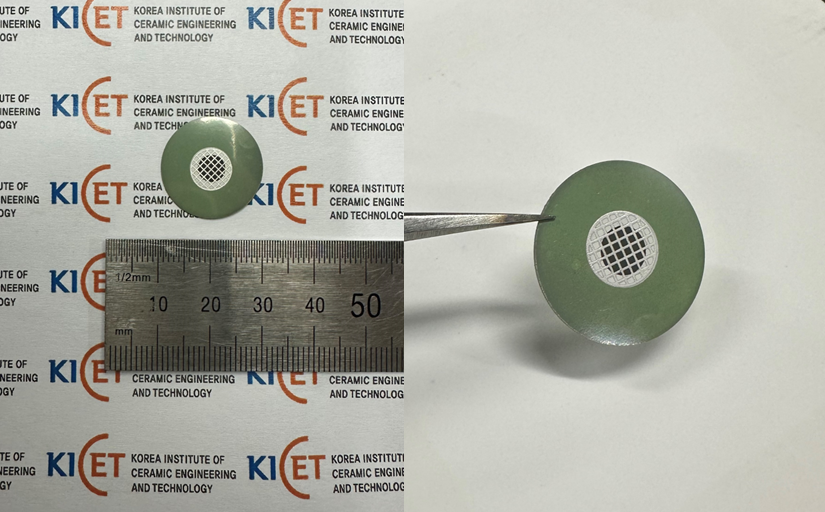
**

Figure S14. The fabricated single-cell configuration with Ni-Y_0.08_Zr_0.92_O_2-δ_ (YSZ)|YSZ|Sm_0.2_Ce_0.8_O_2-δ_|PrBa_0.8_Ca_0.2_Co_2_O_5+δ_. The Ag paste was screen-printed with the grid pattern.


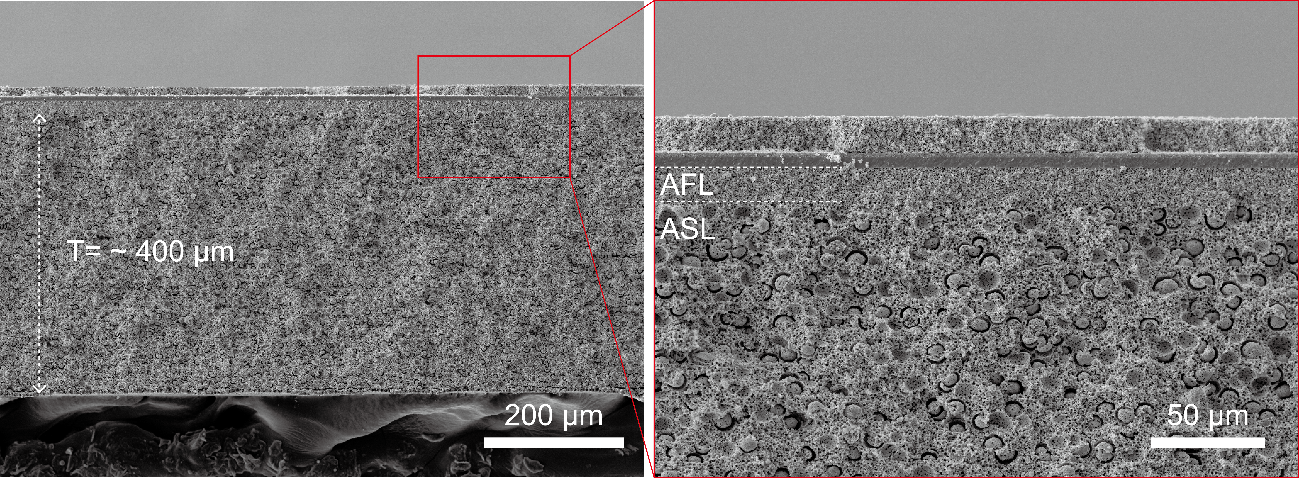


Figure S15. Scanning electron microscopy images of cross-section of the single cell configuration of Ni-Y_0.08_Zr_0.92_O_1.92-δ_ (YSZ)|YSZ|Sm_0.2_Ce_0.8_O_2-δ_|PrBa_0.8_Ca_0.2_Co_2_O_5+δ_; overall cell configuration and enlarged view highlighting the anode functional layer (AFL) and anode support layer (ASL).


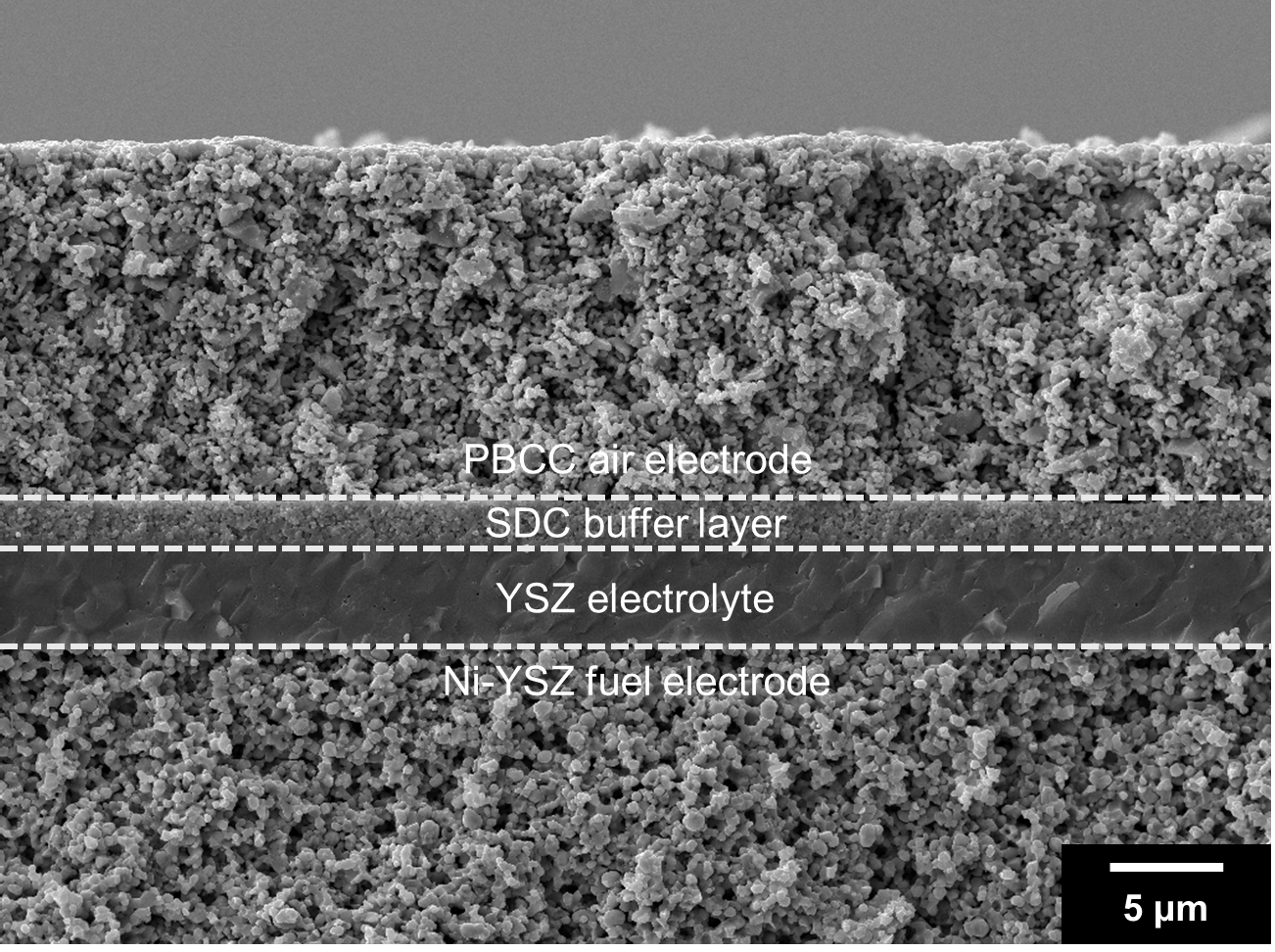


Figure S16. Cross-sectional scanning electron microscopy image of single cell configuration of Ni-Zr_0.92_Y_0.08_O_1.96-δ_ (YSZ)|YSZ|Sm_0.2_Ce_0.8_O_2-δ_ (SDC)|PrBa_0.8_Ca_0.2_Co_2_O_5+δ_ (PBCC) after 10 minutes of alkaline leaching.


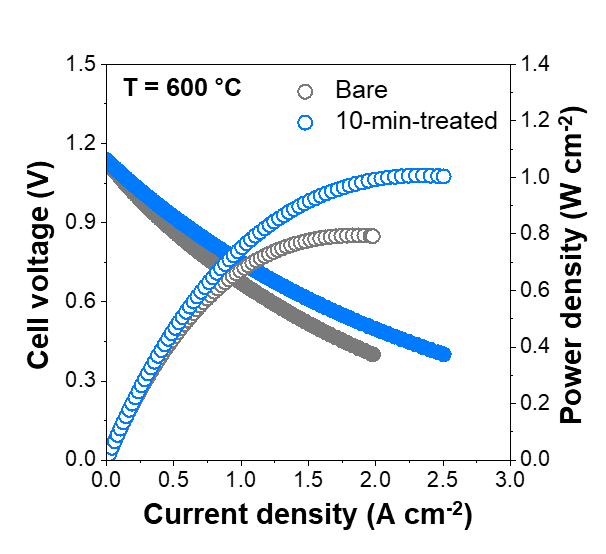


Figure S17. *I-V-P* curves of the single cell with the configuration of Ni-Zr_0.92_Y_0.08_O_1.96-δ_ (YSZ)|YSZ|Sm_0.2_Ce_0.8_O_2-δ_|PrBa_0.8_Ca_0.2_Co_2_O_5+δ_ measured at 600 °C.


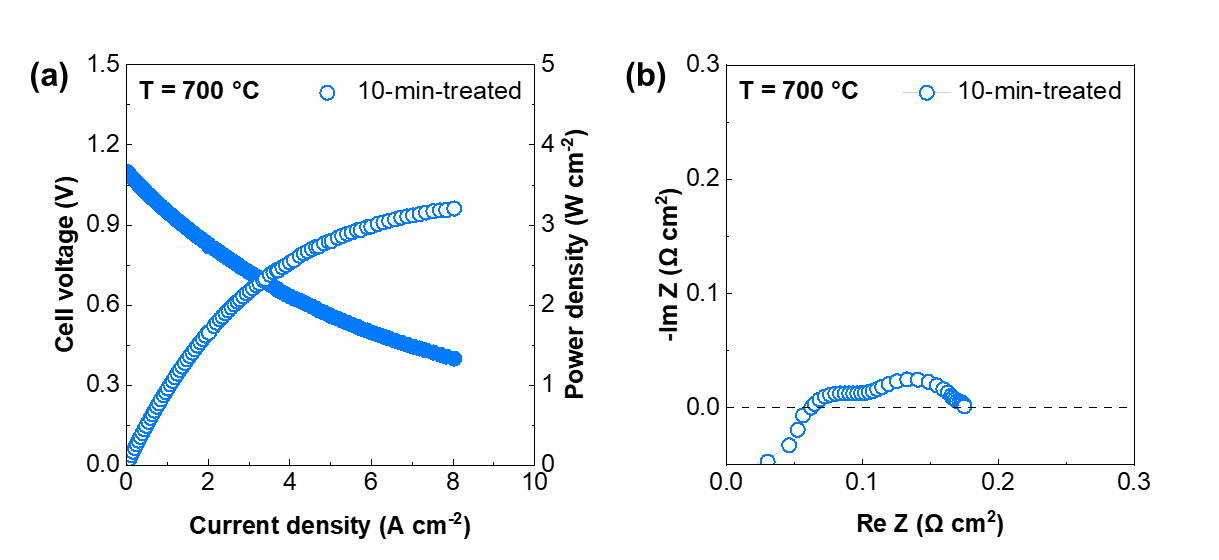


Figure S18. (a) *I-V-P* curves and (b) impedance spectrum of 10-min-treated single cells with the configuration of Ni-Zr_0.92_Y_0.08_O_1.96-δ_ (YSZ)|YSZ|Sm_0.2_Ce_0.8_O_2-δ_|PrBa_0.8_Ca_0.2_Co_2_O_5+δ_ at 700 °C.


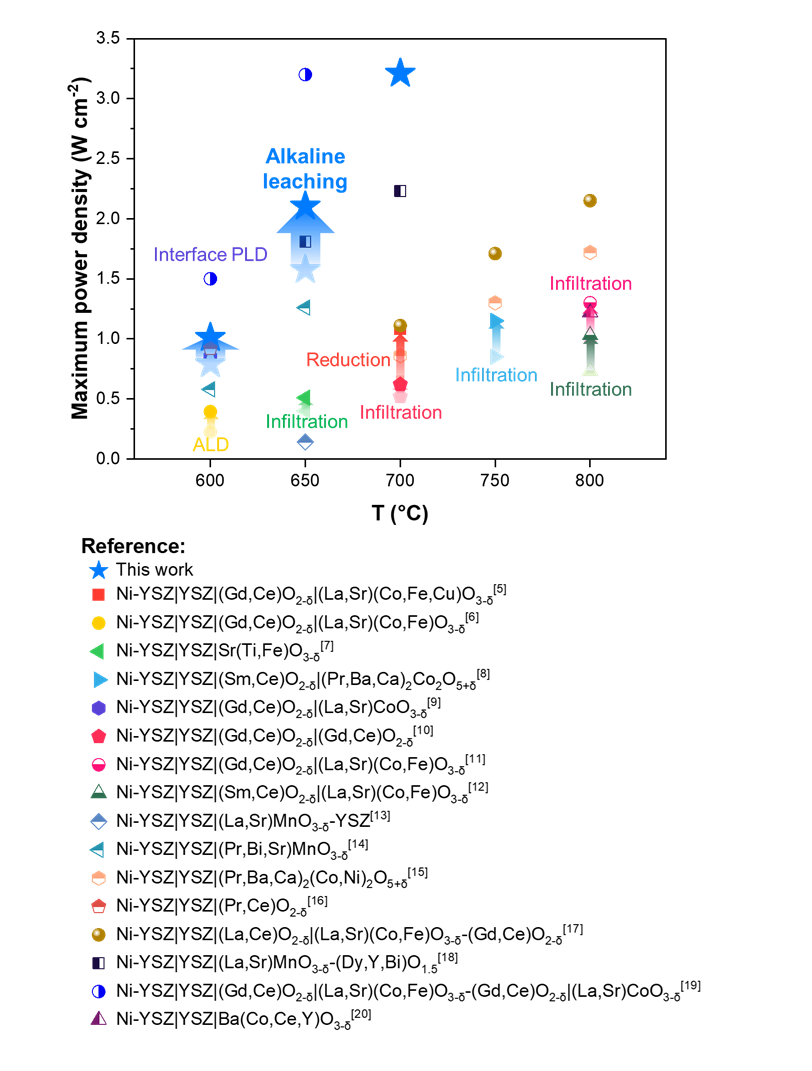


Figure S19. Maximum peak power densities of Ni-(Y,Zr)O_2-δ_ (Ni-YSZ)-based single cells compared to previously reported values^[5-20]^ and this work.


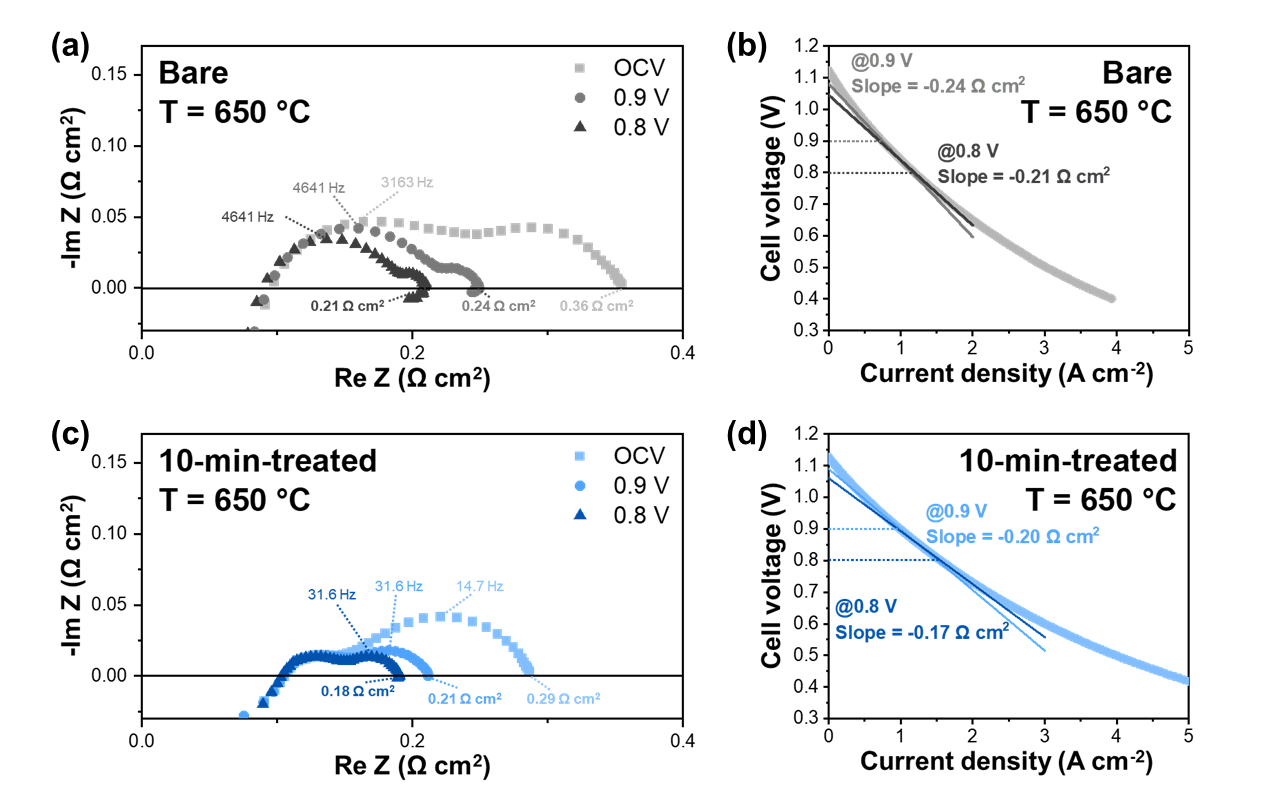


Figure S20. Electrochemical characterization of the single cell with the configuration of Ni-Y_0.08_Zr_0.92_O_1.92-δ_ (YSZ)|YSZ|Sm_0.2_Ce_0.8_O_2-δ_|PrBa_0.8_Ca_0.2_Co_2_O_5+δ_. (a, c) Impedance spectra at OCV and under load of 0.9 and 0.8 V of bare and 10-min-treated cells at 650 °C; (b, d) *I-V* curves with tangents and slopes at 0.9 and 0.8 V.


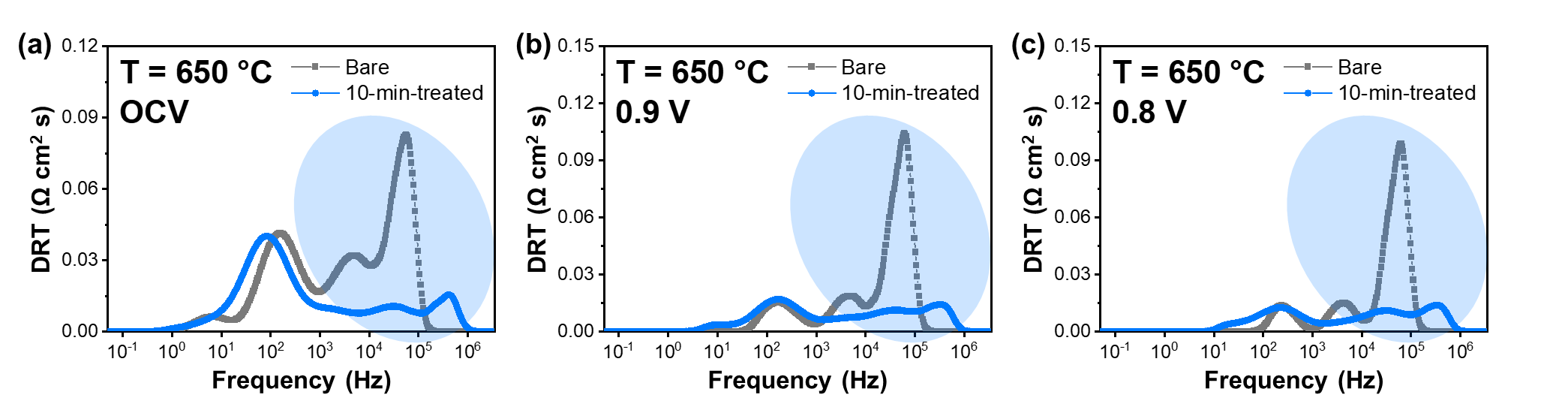


Figure S21. Distribution of relaxation time analysis results of the bare and 10-min-treated single cell with the configuration of Ni-Y_0.08_Zr_0.92_O_1.92-δ_ (YSZ)|YSZ|Sm_0.2_Ce_0.8_O_2-δ_|PrBa_0.8_Ca_0.2_Co_2_O_5+δ_ at 650 °C under (a) OCV, (b) 0.9 V, and (c) 0.8 V.


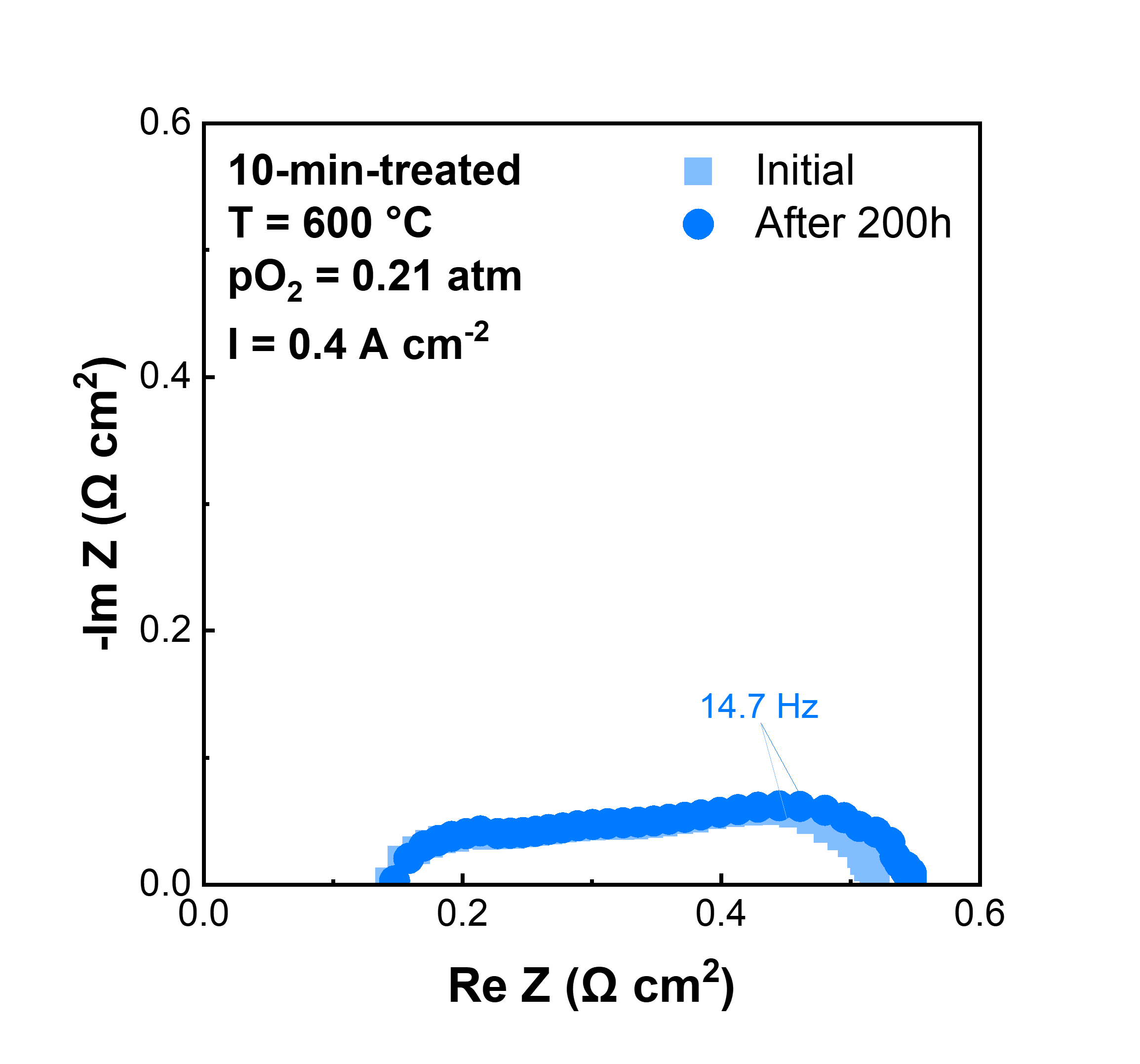


Figure S22. Impedance spectra of the 10-min-treated single-cell configuration of Ni-Zr_0.92_Y_0.08_O_1.96-δ_ (YSZ)|YSZ|Sm_0.2_Ce_0.8_O_2-δ_|PrBa_0.8_Ca_0.2_Co_2_O_5+δ,_ comparing the initial and after 200 hours under 600 °C and 0.4 A cm^-2^.

**References**

[1] Y. Namgung, J. Hong, A. Kumar, D.-K. Lim, S.-J. Song, *Appl. Catal. B* **2020**, *267*, 118374.

[2] H. J. Choi, K. Bae, S. Grieshammer, G. D. Han, S. W. Park, J. W. Kim, D. Y. Jang, J. Koo, J.-W. Son, M. Martin, J. H. Shim, *Adv. Energy Mater.* **2018**, *8*, 1802506.

[3] Y. Chen, A. Hinerman, L. Liang, K. Gerdes, S. P. Navia, J. Prucz, X. Song, *J. Power Sources* **2018**, *405*, 45–50.

[4] D. H. Kim, S. Yang, D.-H. Kwon, H.-I. Ji, J.-W. Son, J. H. Shim, *Int. J. Energy Res.* **2022**, *46*, 12467–12475.

[5] S. Jeon, W.-G. Jung, H. Bae, S. Ahn, B. Koo, W. Yu, S. Kim, D. Oh, U. Kim, S. A. Barnett, J. Seo, B.-J. Kim, W. Jung, *Adv. Mater.* **2024**, *36*, 2404103.

[6] H. J. Choi, K. Bae, S. Grieshammer, G. D. Han, S. W. Park, J. W. Kim, D. Y. Jang, J. Koo, J.-W. Son, M. Martin, J. H. Shim, *Adv. Energy Mater.* **2018**, *8*, 1802506.

[7] D.-Y. Kim, C.-H. Park, B.-K. Park, *J Electrochem. Soc.* **2024**, *171*, 034504.

[8] Y. Chen, S. Yoo, W. Zhang, J. H. Kim, Y. Zhou, K. Pei, N. Kane, B. Zhao, R. Murphy, Y. Choi, M. Liu, *ACS Catal.* **2019**, *9*, 7137-7142.

[9] D. H. Kim, S. Yang, D.-H. Kwon, H.-I. Ji, J.-W. Son, J. H. Shim, *Int. J. Energy Res.* **2022**, *46*, 12467–12475.

[10] Z. Zheng, J. Jing, H. Yu, Z. Yang, C. Jin, F. Chen, S. Peng, *ACS Sustainable Chem. Eng.* **2022**, *10*, 6817-6825.

[11] J. Huang, Q. Liu, S. P. Jiang, L. Zhao, N. Ai, X. Wang, Y. Shao, C. Guan, H. Fang, Y. Luo, K. Chen, *Appl. Catal. B* **2023**, 321, 122080.

[12] M. Nadeem, B. Hu, C. Xia, *Int. J. Hydrogen Energy* **2018**, *43*, 8079-8087.

[13] S. Nam, J. Kim, H. Kim, S. Ahn, S. Jeon, Y. Choi, B.-K. Park, W. Jung, *Adv. Mater.*  **2024**, *36*, 2307286.

[14] K. T. Bae, I. Jeong, D. Kim, H. Yu, H.-N. Im, A. Akromjon, C.-W. Lee, K. T. Lee, *Chem. Eng. J.* **2023**, *461*, 142051.

[15] D. Zeng, F. Zhu, K. Xu, Y. Xu, Y. Chen, *Adv. Funct. Mater.* **2025**, 2505591.

[16] H. G. Seo, D. H. Kim, J. Seo, S. J. Jeong, J. Kim, H. L. Tuller, J.-W. Son, W. Jung, *Adv. Energy Mater.* **2022**, *12*, 2202101.

[17] X. D. Nguyen, S. W. Lee, S. J. Kim, J. Park, B. Koo, S. H. Lee, S. Lee, H. T. Lim, J. T. S. Irvine, T. H. Shin, *Adv. Sci.* **2024**, 11, 2402348.

[18] B.-H. Yun, K. J. Kim, D. W. Joh, M. S. Chae, J. J. Lee, D. Kim, S. Kang, D. Choi, S.-T. Hong, K. T. Lee, *J. Mater. Chem. A* **2019**, *7*, 20558-20566.

[19] K. Develos-Bagarinao, T. Ishiyama, H. Kishimoto, H. Shimada, K. Yamaji, *Nat. Commun.* **2021**, *12*, 3979.

[20] H. Yang, Y. Zhang, Z. Liu, C. Hu, J. Li, H. Liao, M. Shao, M. Ni, B. Chen, Z. Shao, H. Xie, *Nat. Commun.* **2025**, *16*, 3154.
